# Lipoprotein Corona Reshapes the Internal Structure of Lipid Nanoparticles

**DOI:** 10.1021/acsnano.6c09400

**Published:** 2026-09-02

**Authors:** Lucrezia Caselli, Marta Rojas-Rodríguez, Valentina Pacciani, Martino Calamai, Roberto Frigerio, Andrea Zendrini, Gennaro Sanità, Emanuela Esposito, Anna Leung, Hanna Wacklin-Knecht, Ben Humphreys, Paolo Arosio, Jacopo Cardellini, Debora Berti

**Affiliations:** † Department of Chemistry “Ugo Schiff”, University of Florence, 50019 Sesto Fiorentino, Florence, Italy; ‡ CSGI, Center for Colloid and Surface Science, 50019 Sesto Fiorentino, Florence, Italy; § European Laboratory for Non-Linear Spectroscopy, Via Nello Carrara 1, 50019 Sesto Fiorentino, Italy; ∥ National Institute of Optics—National Research Council, Via Nello Carrara 1-I, 50019 Sesto-Fiorentino, Firenze, Italia; ⊥ ETH Zürich, Department of Chemistry and Applied Biosciences, Institute for Chemical and Bioengineering, 8093 Zürich, Switzerland; # EYE-Lab, Institute of Applied Sciences and Intelligent Systems, National Research Council, Via Pietro Castellino 111, 80131 Napoli, Italy; ∇ European Spallation Source ERIC, P.O. Box 176, SE 22100 Lund, Sweden; ○ Institut Laue-Langevin, 71 Avenue des Martyrs, CS20156, 38042 Grenoble, France

**Keywords:** lipid nanoparticles, lipoproteins, biomolecular corona, liquid crystalline lipid nanoparticles, lipid exchange, structural remodeling

## Abstract

Lipid nanoparticles (LNPs) are central to nanomedicine, yet their clinical translation is limited by the difficulty of predicting structure-bioactivity relationships. Although the biomolecular corona is known to define the nanoparticle biological identity by modifying surface properties, its effect on internal nanoparticle organization remains largely unexplored. Since the internal lipid organization of LNPs governs key properties, including stability, cargo protection, internalization and intracellular trafficking, understanding this coupling is critical to correlate synthetic design to therapeutic outcome. Here, combining structural and mechanistic evidence, we show that plasma lipoproteins engage in molecular-scale lipid exchange with the LCNP membrane, rather than persisting as an adsorbed layer of intact particles, actively remodeling the internal nanoparticle structure. Using model lipid liquid-crystalline nanoparticles with well-defined cubic and inverse hexagonal phases, we systematically investigate how lipoproteins modulate LNP structure. Combining fluorescence nanoparticle tracking, synchrotron small-angle X-ray scattering, cryo-electron microscopy, and neutron reflectometry with isotopic contrast matching, we provide evidence of lipoprotein-driven lipid transfer and phase reorganization at the nanoscale. We observe a phase-selective response: inverse hexagonal LNPs remain structurally stable despite lipid exchange, whereas cubic LNPs undergo pronounced remodeling, including significant change in size and lattice ordering. These transformations correlate with distinct cellular uptake profiles. Our findings suggest that in soft lipid nanoparticles, the biological identity commonly described as a biomolecular corona is not merely a surface event but an interface-mediated process that reshapes the nanoparticle internal structure, with direct implications for next-generation nanomedicine design.

## Graphical abstract

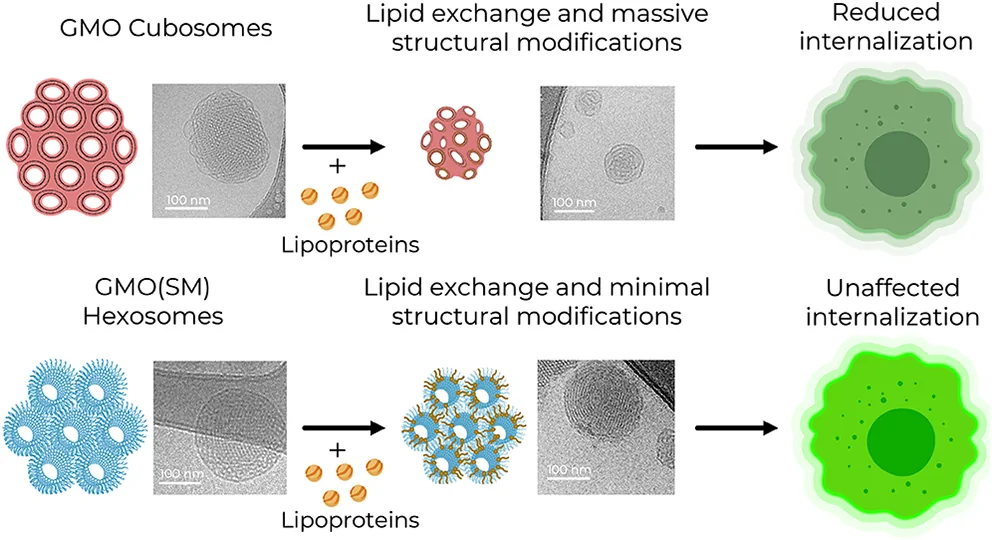

## Introduction

Over the past decade, lipid nanoparticles (LNPs) have moved to the forefront of nanomedicine, mainly through their clinical deployment for mRNA-based COVID-19 vaccines.^1–3^ This success has established lipid-based nanocarriers as versatile delivery system for a broad range of cargos, from small hydrophobic drugs to large biomacromolecules, such as RNA and proteins.^4,5^ Despite this progress, the in vivo performance of LNPs often deviates from in vitro observations, limiting our ability to control therapeutic efficacy based on particle composition and structure.^6,7^

A major contribution to this discrepancy is the complex biological environment encountered after administration.^8,9^ In biological fluids, LNPs interact with proteins, lipoproteins, and other macromolecules that adsorb onto their surface, forming a biomolecular corona. This corona redefines LNP biological identity and strongly influences cellular uptake pathways, biodistribution, and clearance kinetics. For instance, apolipoproteins contained in plasma lipoproteins, particularly ApoE, can promote receptor-specific uptake of LNPs, thereby modulating tissue tropism.^10–13^ Recent studies also indicate that corona formation may reduce endosomal escape and RNA delivery.^14^ To date, however, these effects have been interpreted from a surface-centric perspective:^15–20^ the corona is viewed as an interfacial shell that modifies charge, receptor recognition, and membrane interactions, while the effects on the internal structure of the LNP remains almost entirely overlooked.

Recent evidence, however, suggests that this view might be incomplete. Sebastiani et al. reported that ApoE-containing coronas can induce lipid redistribution within LNPs, indicating that corona components may dynamically interact with the lipid core.^21^ This raises the broader possibility that corona might remodel the internal lipid organization.

This question is particularly relevant because LNPs are soft, dynamic assemblies, whose structure is determined by a delicate balance of intermolecular interactions.^22^ Unlike rigid inorganic nanoparticles,^23,24^ LNPs can exchange molecular components with their environment. Such processes, including lipid transfer between LNPs and biomembranes, are known to modulate membrane curvature, fusion, and structural remodeling in biological systems.^20,25^ Thus, biomolecular association at the LNP interface may not only alter surface recognition but also drive molecular exchange and compositional redistribution within the nanoparticle core. Whether these phenomena can directly reshape the internal architecture of LNPs remains unknown. This is a critical question, as LNPs’ biological performance is tightly coupled to the internal nanostructure, which governs colloidal stability, cargo protection, membrane fusion processes, and intracellular trafficking.^26–29^

Here, we address this gap using lipid liquid-crystalline nanoparticles (LCNPs) as structurally defined model systems. LCNPs offer precisely engineered internal nanostructure, such as bicontinuous cubic (*Pn*3*m* or *Im*3*m*) and inverse hexagonal (H_II_) architectures. Their ordered internal lattices, tunable membrane properties, and well-defined phase behavior endow LCNPs with unique functional capabilities as next-generation lipid nanocarriers.^30–34^ Importantly, the periodicity of these internal structures generates intense, structure-specific scattering features (i.e., Bragg peaks) enabling quantitative monitoring of internal reorganization by scattering-based techniques.^35,36^ LCNPs therefore provide structurally diagnostic model platforms for determining whether interactions with endogenous biomolecules initiated at the nanoparticle interface propagate into the internal lipid matrix. Establishing this mechanism in simplified, well-defined systems provides a foundation for subsequent studies of more compositionally complex and clinically relevant LNP and liposomal formulations, in which comparable remodeling may be more difficult to resolve.

Among the biomolecules encountered in vivo, we focus on plasma lipoproteins, identified as major constituents of LNPs corona, with apolipoproteins (e.g., ApoE, ApoB) affecting cellular uptake and biodistribution.^26^ These nanoscale lipid–protein complexes transport triglycerides and cholesterol and are structurally composed of a hydrophobic core surrounded by a phospholipid monolayer enriched in cholesterol and apolipoproteins.^37^ Besides receptor-mediated interactions, lipoproteins can exchange lipids with cell membranes via passive, receptor-independent mechanisms that alter membrane composition and mechanics.^38–40^

By combining fluorescence-based nanoparticle tracking analysis (NTA) with synchrotron small-angle X-ray scattering (SAXS) and cryogenic electron microscopy (cryo-EM), we show how lipoprotein association drives structural reorganization of LCNPs. Furthermore, using neutron reflectometry with isotopic contrast matching on model LCNP membranes, we demonstrate that lipoprotein-driven lipid transfer is the molecular mechanism underlying nanoparticle remodeling.

We show that lipoproteins induce phase-dependent reorganization of LCNPs. While hexagonal LCNPs maintain structural integrity despite measurable lipid exchange, cubic LCNPs undergo extensive reorganization, accompanied by the loss of lattice ordering and changes in particle size. These transformations correlate with altered cellular uptake, linking nanoscale remodeling to biological function.

Collectively, our results demonstrate that lipoprotein association with LNPs extends beyond surface modification to drive LNP internal structural reprogramming. This previously overlooked coupling between the corona and internal organization defines a new level of complexity for understanding, and ultimately controlling, the behavior of lipid nanocarriers in biological environments.

## Results

### LCNPs Characterization

To investigate the impact of lipoproteins on the internal organization of lipid nanoparticles, we employed two glycerol monooleate (GMO)-based liquid crystalline nanoparticles (LCNPs). These systems combine simple and defined composition with highly ordered architectures that generate distinct Bragg reflections, providing a direct readout of internal symmetry and lattice parameters through scattering techniques. All formulations were prepared in 10 mM Tris buffer at pH 7.5 ± 0.1 to ensure physiological relevance and colloidal stability.

The first formulation consists of GMO LCNPs, sterically stabilized with 10 wt % Pluronic F-127. GMO is an FDA-approved monoglyceride, classified as “generally recognized as safe” (GRAS) for use in food and pharmaceuticals, widely employed in lipid-based nanocarriers. As a representative inverse-curvature lipid, GMO exhibits rich polymorphism in aqueous environments, forming lyotropic phases such as bicontinuous cubic and inverse hexagonal structures, depending on environmental conditions and molecular inclusions.^41,42^ Due to this structural versatility and compositional simplicity, GMO-based LCNPs are broadly studied in drug delivery to encapsulate both hydrophilic and hydrophobic cargos, including nucleic acids.^43^ As such, they provide a structurally defined and relevant model scaffold for LNP formulations.

The second formulation is a binary mixture of GMO and the ionizable lipid SM-102 (10 mol %, hereafter GMO­(SM)), stabilized with 10 wt % Pluronic F-127. SM-102 is the ionizable lipid used in Spikevax, where its pH-dependent protonation contributes to endosomal escape. Its incorporation into the GMO matrix provides a simplified, structurally defined platform containing a clinically relevant ionizable lipid, without reproducing the complete composition or internal architecture of the corresponding clinical formulation.

LCNPs were prepared by microfluidic ethanol injection,^44,45^ yielding nanometric-sized nanoparticles. DLS measurements indicate average hydrodynamic diameters of approximately 255 nm for GMO and 270 nm for GMO­(SM) (PDI = 0.25 and 0.24, respectively; Figure S2 and Table S1, Supporting Information). At pH 7.5, GMO LCNPs exhibit a negative ζ-potential (−21 ± 2 mV), commonly attributed to residual free fatty acids present in commercial GMO.^46^ In contrast, GMO­(SM) LCNPs display near-neutral surface charge (+5 ± 2 mV), reflecting partial protonation of SM-102 under these conditions, which compensates for the negative contribution of fatty acids contained in GMO.

The morphology of LCNPs was visualized through cryo-EM. Representative cryo-EM micrographs are shown in Figure 1A. Both formulations exhibit well-defined, roughly spherical nanoparticles with an ordered, periodic internal nanostructure. The particle core is surrounded by a multilayer-like interfacial organization, in agreement with previous reports on similar systems^26,47^ (see Figures S3 and S4 of Supporting Information for additional images).

**Figure 1:**
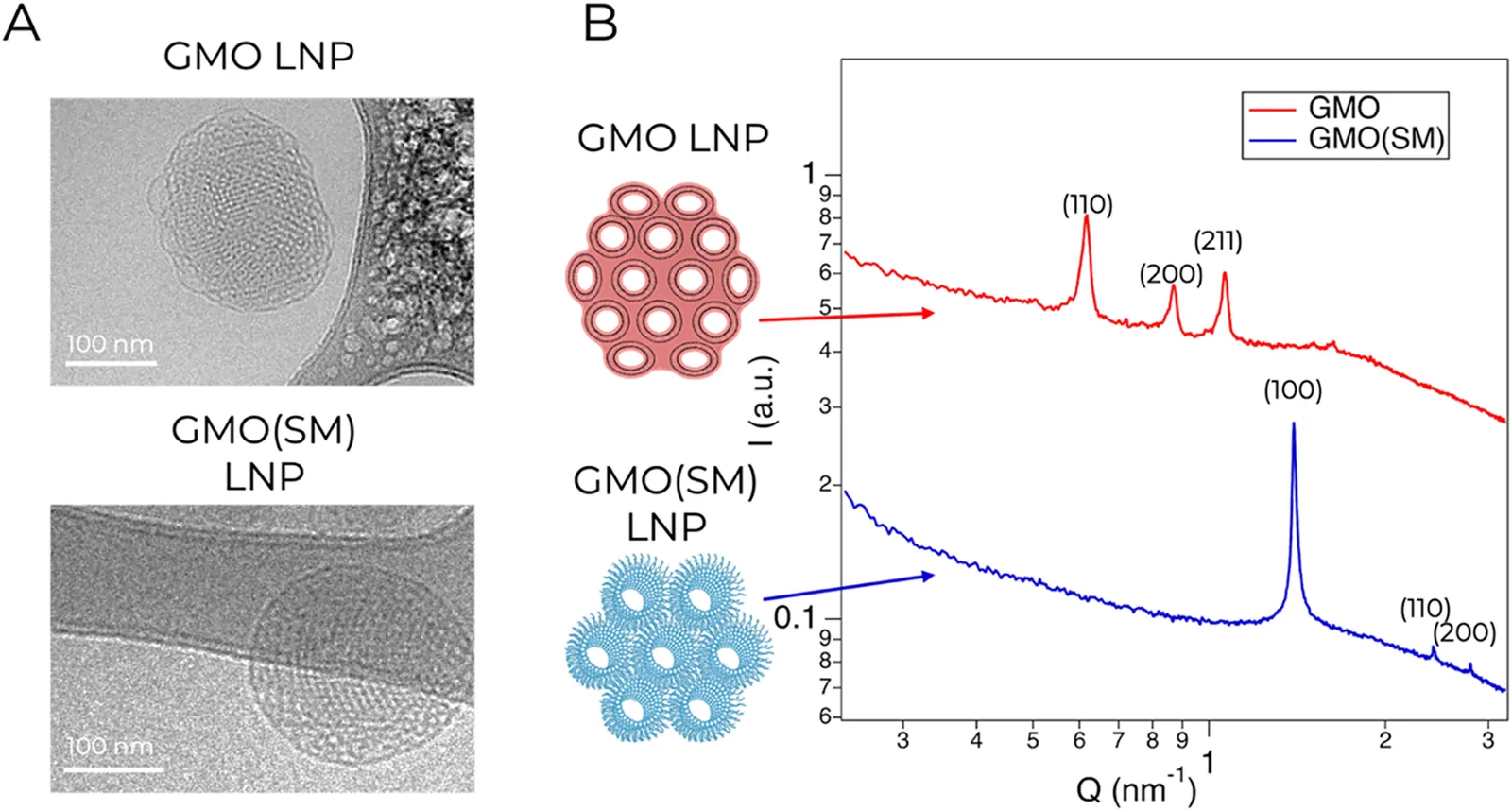
Representative Cryo-TEM micrographs of (A) GMO LCNPs (top) and GMO­(SM) LCNPs (bottom), in Tris buffer, 10 mM, pH 7.5 ± 0.1. (B) SAXS profiles and corresponding structural models of 12.5 mg/mL (total lipid concentration) GMO and GMO­(SM) LCNPs. The SAXS profiles are vertically offset along the *y*-axis for clarity. Bragg peaks are indexed with the corresponding Miller indices of the Pn3m (GMO LCNPs) and H_II_ (GMO­(SM) LCNPs) phases. All measurements were performed in Tris buffer, 10 mM, pH 7.5 ± 0.1 at 25 °C.

Furthermore, the internal structural organization of the LCNPs was monitored at the ensemble-averaged level by synchrotron SAXS. SAXS profiles for both formulations are shown in Figure 1B. GMO LCNPs exhibit three well-defined and intense Bragg reflections that can be indexed to the (110), (200), and (211) crystallographic planes, consistent with an inverse *Im*3*m* bicontinuous cubic phase, where a continuous lipid bilayer separates two interpenetrating but nonintersecting aqueous channel networks. SAXS data analysis provided a lattice spacing (*d*) of 14.3 ± 0.1 nm and a water channel radius (*r*_w_) of 2.6 ± 0.1 nm, in line with previously reported cubic GMO-based nanoparticle systems^48^ (see Methods section for SAXS data analysis). The SAXS pattern of GMO­(SM) LCNPs displays three characteristic reflections indexed to the (100), (110), and (200) planes, indicative of an inverse hexagonal (H_II_) phase with d 5.2 ± 0.1 nm and *r*_w_ of 0.8 ± 0.1 nm. This liquid crystalline phase is composed of inverse cylindrical lipid micelles, packed into a hexagonal lattice. The transition from an inverse cubic to an inverse hexagonal phase upon incorporation of 10 mol % SM-102 can be rationalized considering the molecular geometry of SM-102.^41^ Relative to GMO, SM-102 features a smaller headgroup and bulky double hydrocarbon chains, corresponding to a higher intrinsic critical packing parameter (CPP) and a stronger preference for negative curvatures. Incorporation of SM-102 therefore biases the overall packing toward higher negative curvature, favoring inverse hexagonal organization. This phase behavior is consistent with previous reports on SM-102–containing GMO LCNPs.^49,50^

Overall, the well-defined internal structure of both formulations provides two distinct, robust experimental platforms to systematically investigate how interactions with lipoproteins impact lipid nanoparticle organization.

### Interaction of LCNPs with Lipoproteins

In biological fluids, LCNPs are exposed to several classes of circulating lipoproteins that compete for adsorption at the nanoparticle interface. In this work, we selected three of the most abundant lipoprotein components of human serum,^51^ i.e., LDL, HDL, and VLDL and investigated their interactions with LCNPs. LDL, HDL, and VLDL were characterized in 10 mM Tris buffer (pH 7.5 ± 0.1) at 25 °C (see Figure S5) prior to use. The characteristic lipid and apolipoprotein composition of human-derived lipoproteins is summarized in Table S2 (Supporting Information).

To quantify the association of lipoprotein-derived material with LCNPs, we employed fluorescence nanoparticle tracking analysis (f-NTA). By tracking individual particle trajectories with two fluorescence detector channels, f-NTA enables direct and quantitative assessment of colocalization of LCNPs and lipoproteins, providing robust evidence of lipoprotein corona formation.

LCNPs were labeled with the fluorescent lipid probe β-Bodipy FL C12 (λ_ex_ = 500 nm and λ_em_ = 510 nm), whereas LDL, HDL, and VLDL particles were labeled with Alexa Fluor 647 NHS ester (λ_ex_ = 647 nm and λ_em_= 670 nm), which covalently reacts with primary amino groups on apolipoproteins exposed at the lipoproteins surface. The free dye in excess was removed by size-exclusion chromatography (SEC) following previously reported protocols (see Materials and Methods section and Supporting Information).^52,53^

GMO cubosomes or GMO­(SM) hexosomes, at a fixed lipid concentration (0.25 mg mL^–1^), were incubated with ternary HDL/LDL/VLDL lipoprotein mixtures at 25 °C in 10 mM Tris buffer (pH 7.5 ± 0.1) for 2 h. The three lipoprotein classes were combined at equal concentrations (1/1/1 wt), and the total lipoprotein concentration in the incubation samples was fixed at 0.012 mg mL^–1^, corresponding to a lipoprotein/LCNP weight ratio of 5%.

Within each ternary mixture, only one lipoprotein class was fluorescently labeled, while the other two remained unlabeled. Accordingly, samples containing labeled HDL together with unlabeled LDL and VLDL were defined as “**mix 1”**, whereas the complementary conditions with selectively labeled LDL or VLDL were defined as “**mix 2”** and “**mix 3”**, respectively (Figure 2A,B). This experimental design enables the selective tracking of each individual lipoprotein class and allows direct comparison of their relative propensities to associate with LCNPs.

**Figure 2:**
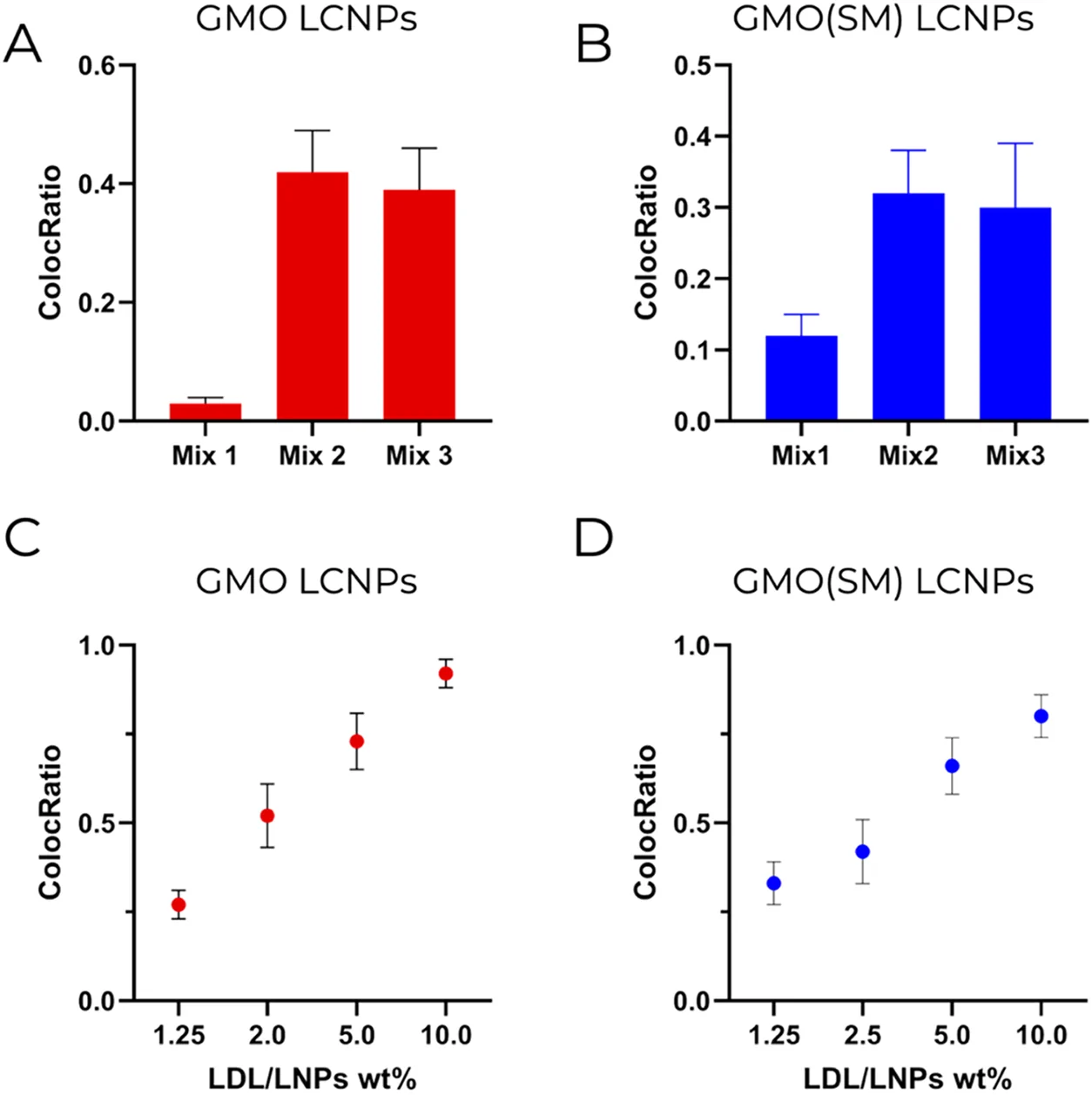
Colocalization ratio obtained from fluorescence NTA measurements of β-Bodipy-labeled (A) GMO and (B) GMO­(SM) LCNPs after 2 h incubation with Alexa Fluor 647-labeled lipoprotein mixtures (lipoproteins/LCNPs, 5 wt %). In mix 1, HDL are labeled while LDL and VLDL are unlabeled; in mix 2, LDL are labeled while HDL and VLDL are unlabeled; in mix 3, VLDL are labeled while HDL and LDL are unlabeled. Colocalization ratios for BODIPY-labeled (C) GMO and (D) GMO­(SM) LCNPs incubated for 2 h with Alexa Fluor 647-labeled LDL as a function of the LDL/LCNP weight ratio. Measurements were performed in triplicate.

The association of the different lipoprotein classes to LCNP was quantified through the colocalization ratio (“colocRatio”; Figure 2A,B). This ratio is defined as the number of detected particles positive for both β-Bodipy (staining LCNPs), and Alexa Fluor 647 (staining lipoproteins), divided by the total number of β-Bodipy-positive particles. A colocRatio of 1 therefore indicates complete association of all detected LCNPs with fluorescently labeled lipoproteins, whereas lower values reflect a progressively smaller fraction of LCNPs associated with lipoproteins.

The results (Figure 2A,2B) reveal a pronounced disparity in lipoprotein association. HDL display a significantly lower colocalization with both GMO and GMO­(SM) LCNPs compared to LDL and VLDL. The same qualitative ranking was observed for both LCNP formulations under the equimass competitive conditions tested, suggesting that lipoprotein class is an important determinant of association in these systems. Given the lower abundance of VLDL relative to LDL in healthy plasma,^37^ these findings suggest that LDL may contribute substantially to the lipoprotein-derived material associated with these LCNPs under physiological conditions. Accordingly, LDL was selected as the representative lipoprotein for the mechanistic studies described in the following sections.

Having established preferential association under competitive conditions, we investigated LDL–LCNP interactions as a function of lipoprotein concentration. GMO cubosomes and GMO­(SM) hexosomes (0.25 mg mL^–1^) were incubated for 2 h with increasing LDL concentrations corresponding to LDL/LCNP weight ratios of 1.25%, 2.5%, 5%, and 10% in 10 mM Tris buffer (pH 7.5 ± 0.1) at 25 °C. As shown in Figure 2C,D, colocalization between LCNPs and LDL is detectable already at the lowest LDL/LCNP ratio for both formulations, and increases monotonically with LDL concentration, approaching near-complete colocalization at LDL/LCNP 10 wt %, indicating that all LCNPs detected are associated with LDL. Notably, no statistically significant differences in colocalization efficiency are observed between GMO cubosomes and GMO­(SM) hexosomes over the entire concentration range.

Importantly, the LDL/LCNP ratios here explored remain substantially lower than those realistically encountered under physiological conditions, suggesting that in biological fluids LCNPs are likely exposed to an excess of lipoproteins and therefore rapidly become saturated with adsorbed species.

Additionally, both GMO cubosomes and GMO­(SM) hexosomes associated efficiently not only with LDL, but also with HDL and VLDL when each lipoprotein class was tested individually, i.e., in the absence of competition among lipoprotein classes (Figure S6). These results show that all major lipoprotein classes are, in principle, able to associate with LCNPs and contribute lipoprotein-derived material to the biological identity of the nanoparticles.

### LCNP Structural Remodeling Induced by Lipoproteins

To assess possible LCNP remodeling induced by lipoprotein association, we performed synchrotron SAXS. GMO cubosomes and GMO­(SM) hexosomes (12.5 mg mL^–1^) were incubated for 2 h with increasing LDL concentrations, corresponding to LDL/LCNP weight ratios ranging from 0 to 5 wt % (see Method seection). Control measurements of LDL alone were performed to evaluate their contribution to the scattering signal (see Figure S7). Based on these controls, the LDL/LCNP ratio was limited to a maximum of 5 wt % to prevent the scattering signal of the lipoproteins from masking the structural features of the LCNPs.

As shown in Figure 3A, SAXS profiles reveal a pronounced, LDL concentration-dependent destabilization of the cubic internal architecture of GMO cubosomes. Even at the lowest LDL concentration, a marked reduction in the intensity of the Bragg reflections characterizing the cubic *Im*3*m* phase is observed, indicating loss of structural integrity. At higher LDL concentrations (3.3–5 wt %), the ordered *Im*3*m* cubic lattice is almost completely disrupted, with Bragg peaks becoming barely detectable. This loss of structural order is accompanied by a systematic shift of the reflections toward higher q values, consistent with lattice contraction (from 14.3 ± 0.1 nm to 13.2 ± 0.1 nm).

**Figure 3:**
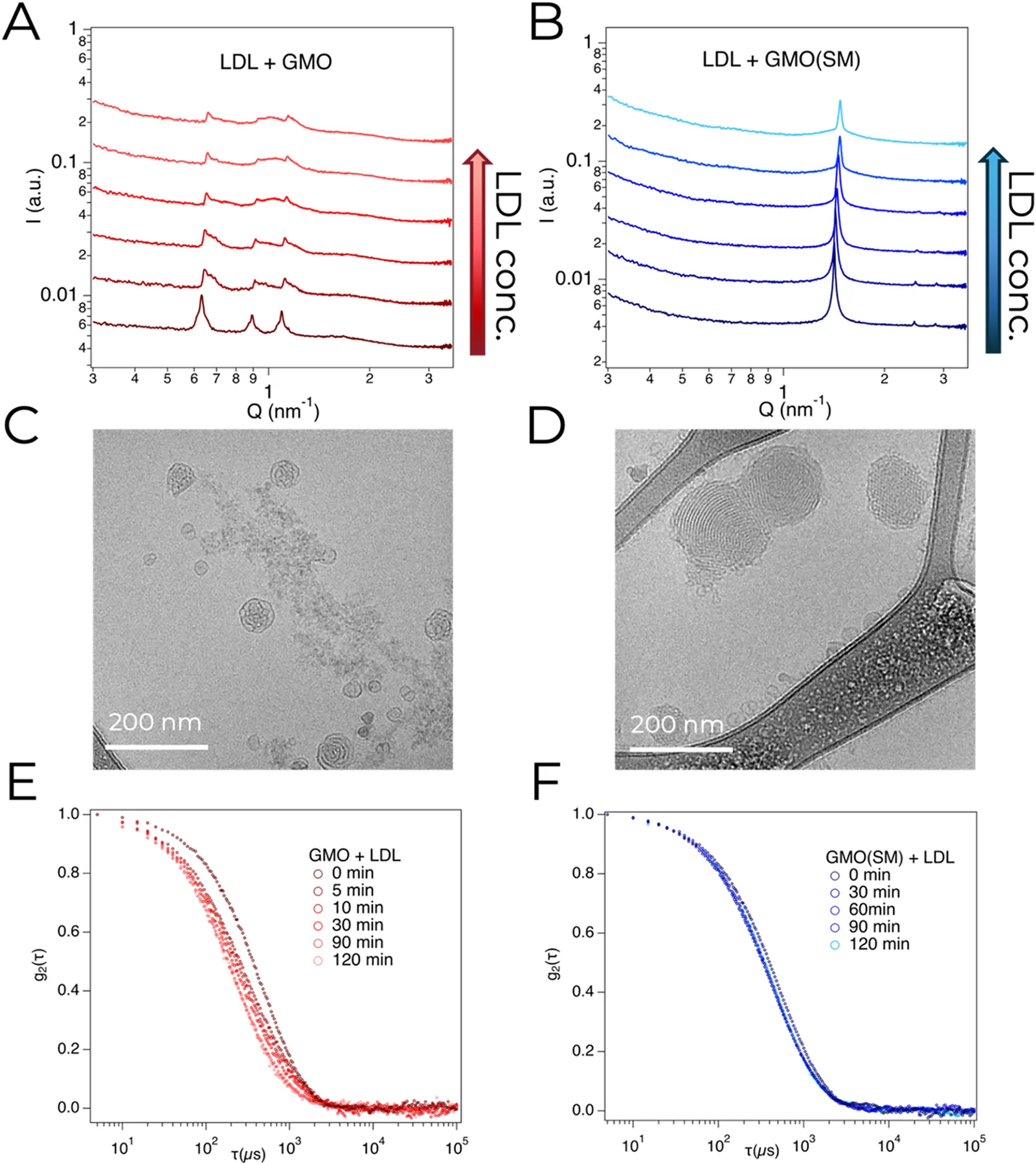
SAXS profiles of (A) GMO and (B) GMO­(SM) LCNPs (12.5 mg/mL) after 2 h of incubation with LDL at different LDL/LCNP weight ratios (0%, 0.7%, 1.2%,2.5%, 3.3%, and 5%). The SAXS profiles are vertically offset along the *y*-axis for clarity. All measurements were performed in 10 mM Tris buffer, pH 7.5 ± 0.1 and 25 °C. Cryo-TEM images of (C) GMO and (D) GMO­(SM) LCNPs after 2 h of interaction with LDL at a LDL/LCNP weight ratio of 5 wt %. Time evolution of DLS autocorrelation functions for (E) GMO and (F) GMO­(SM) LCNPs incubated with LDL (LDL/LCNPs, 5 wt %).

Importantly, structural evolution continues over longer time scales. After 12 h of incubation (see Figure S8), the cubic phase is no longer detectable: Bragg reflections disappear and are replaced by a broad scattering feature centered at *q* ≈ 1.2 nm^–1^, indicative of a disordered or weakly correlated structure.

In contrast, GMO­(SM) hexosomes exhibit markedly higher resistance to LDL-induced perturbations (Figure 3B). Despite comparable levels of LCNPs associated with LDL, as indicated by f-NTA, the characteristic Bragg reflections of the inverse hexagonal (H_II_) phase remain clearly detectable after 2h of incubation even at the highest LDL concentration. Nevertheless, SAXS reveals subtle but systematic structural changes. Specifically, LDL exposure induces a progressive decrease in peak intensity and a shift of the primary hexagonal reflection toward higher q values, corresponding to a contraction of the lattice parameter from 5.2 ± 0.1 nm to 4.9 ± 0.1 nm. After prolonged incubation (12 h, see Figure S7), the hexagonal structure is only partially preserved, with residual Bragg features coexisting with a broad scattering contribution, indicative of partial loss of long-range order. Since hydrophobic inclusions are known to promote more negative membrane curvature in inverse lyotropic liquid-crystalline phases,^54^ which typically results in a contraction of the cubic lattice parameter, these changes may indicate incorporation of hydrophobic molecular components from LDL into the lipid matrix of LCNPs.

To directly visualize the morphological consequences of LDL interaction, we performed cryo-EM measurements. LCNPs (12.5 mg mL^–1^) were incubated with LDL (5 wt %) for 2 h prior to imaging. Representative micrographs of GMO–LDL samples (Figure 3C) reveal pronounced morphological changes compared to native particles (Figure 1A). In particular, the average particle size is significantly reduced, and the characteristic internal cubic organization is largely lost. Only a minor fraction of particles retains residual cubic order (Figure S9), while the majority displays heterogeneous, poorly ordered morphologies, including amorphous aggregates, multilamellar structures, and flower-like, swollen particles consistent with sponge-like (L_3_) phases.^55^

These observations are corroborated by DLS, which shows a progressively faster decay rate of the intensity autocorrelation function, as the incubation time increases (Figure 3E), corresponding to a reduction in hydrodynamic diameter from ∼250 nm to ∼100 nm after 2 h. Together, cryo-EM and DLS indicate that LDL interaction induces a global restructuring of the GMO cubosomes, affecting both internal organization and overall particle size.

In contrast, GMO­(SM) hexosomes display significantly higher morphological stability. Cryo-EM and DLS analyses indicate that both particle size and internal organization are largely preserved upon LDL interaction (Figure 3D,F). However, closer inspection of cryo-EM images reveals increased surface corrugation and the presence of interfacial material, consistent with consistent with lipoprotein-derived components becoming associated with the nanoparticle interface (see Figure S10 for additional images).

The greater structural resilience of GMO­(SM) hexosomes could, in principle, arise from differences in internal liquid-crystalline architecture, lipid composition, and/or surface potential relative to GMO cubosomes (Table S1). To disentangle these contributions, two additional formulations containing lower amounts of SM-102 than GMO­(SM) (i.e., 1 and 2.5 mol % SM-102) were prepared. Both formulations retained the *Im*3*m* cubic phase (Figure S10) while exhibiting near-neutral ζ-potentials of −5 ± 2 and −2 ± 2 mV, respectively, comparable to that of GMO­(SM) hexosomes (+5 ± 2 mV) and significantly less negative to that of GMO cubosomes (−21 ± 2 mV).

Despite their near-neutral surface potentials and the presence of the ionizable lipid, both cubic formulations underwent pronounced LDL-induced remodeling closely resembling that observed for GMO cubosomes (Figure S11). At low LDL/LCNP ratios, the *Im*3*m* reflections broadened and shifted toward higher Q values, whereas at higher ratios the characteristic cubic reflections largely disappeared and were replaced by broad scattering features, indicating extensive loss of long-range cubic order. These results demonstrate that neither near-neutral surface potential nor the presence of the ionizable lipid alone accounts for the greater structural resilience of GMO­(SM) hexosomes. Within the formulation series investigated, they instead identify the internal liquid-crystalline architecture (and the associated membrane packing) as the major determinant of resistance to lipoprotein-induced remodeling.

We next examined whether similar structural effects arise from interactions with other major plasma lipoproteins. We then investigated the structural effect of HDL or VLDL on LCNPs. GMO cubosomes or GMO­(SM) hexosomes were incubated for 2 h with each lipoprotein class at a lipoprotein/LCNP weight ratio of (5 wt %), matching the highest concentration used in the LDL SAXS experiments.

As Figure 4 shows, HDL- and VLDL-treated samples exhibit structural changes closely resembling those observed with LDL. For GMO cubosomes, exposure to either lipoprotein results in near-complete loss of the *Im*3*m* internal cubic phase. In contrast, the inverse hexagonal phase of GMO­(SM) LCNPs remains largely preserved, although the primary reflection systematically shifts toward higher scattering vector (*Q*) values, consistent with lattice contraction.

**Figure 4:**
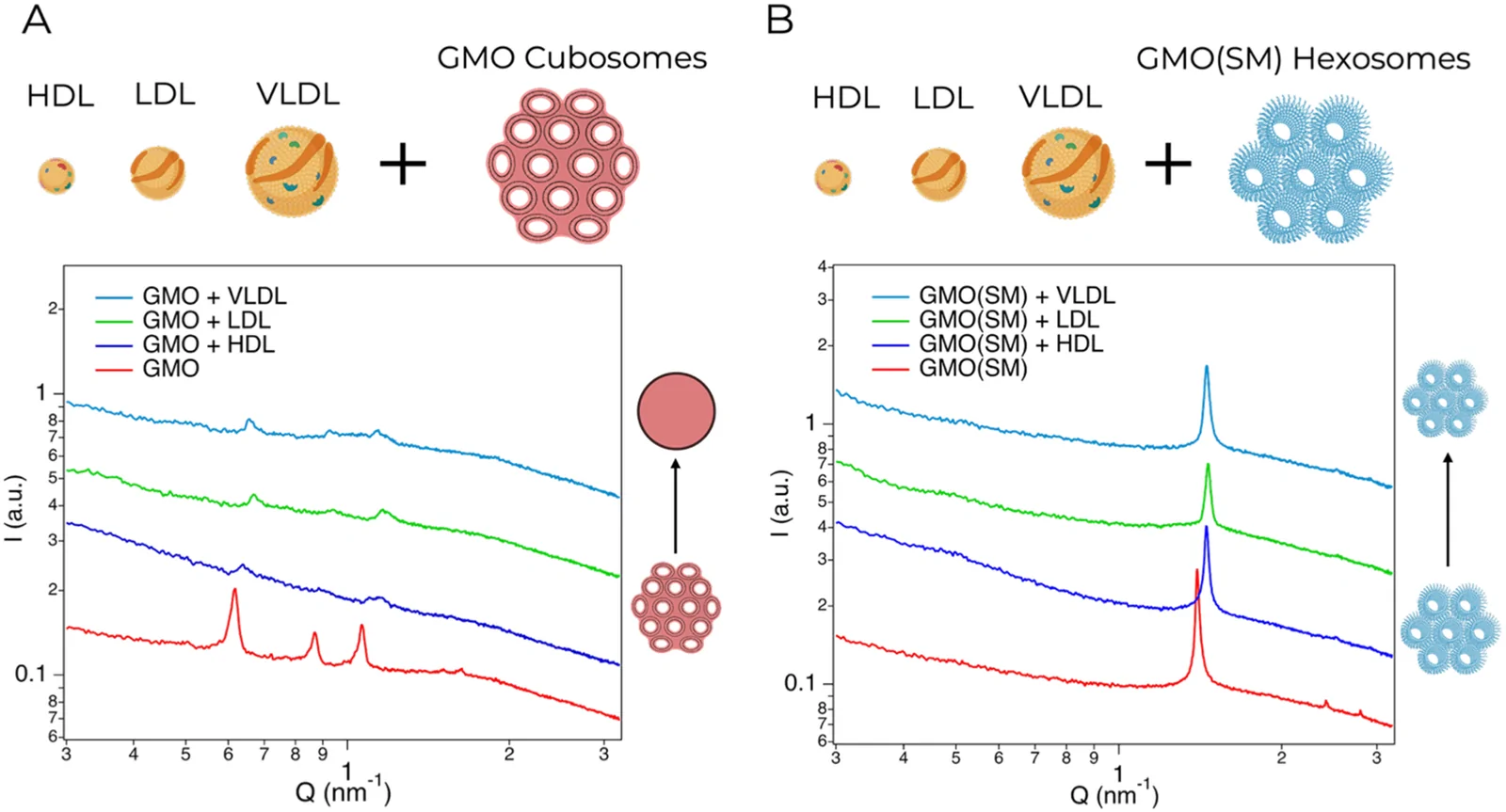
SAXS profiles of (A) GMO and (B) GMO­(SM) LCNPs before and after 2 h of interaction with HDL (blue), LDL (green), and VLDL (light blue) at a lipoprotein/LCNP weight ratio of 5 wt %. The SAXS profiles are vertically offset along the *y*-axis for clarity. All measurements were performed in 10 mM Tris buffer, pH 7.5 ± 0.1 and 25 °C. Figure partially created in BioRender.

The consistency of these structural responses across all lipoprotein classes confirms that LCNP remodeling is not specific to LDL, but rather a general consequence of interactions with lipoproteins. The structural remodeling is induced in both cubic and hexagonal LCNPs, albeit at markedly different extent. Specifically, SAXS analysis shows that, in both systems, remodeling proceeds through lattice contraction, which, in inverse lipid phases, commonly arises from the incorporation of hydrophobic species, that increase negative spontaneous curvature and reduce effective hydration.^41,56^ This suggests that lipoprotein interaction involves material exchange between lipoproteins and LCNPs, possibly including the transfer of hydrophobic lipids such as cholesterol and triglycerides, which are abundant in lipoproteins (Table S2). Incorporation of these components into the LCNP membrane might perturb the curvature–composition balance that stabilizes the internal nanostructure, inducing remodeling at the whole particle level.

To isolate the structural contribution of key LDL lipid components, we performed complementary SAXS experiments using protein-free LDL-inspired lipid mimics composed of POPC (1-palmitoyl-2-oleoyl-*sn*-glycero-3-phosphocholine), triolein, cholesteryl oleate, and cholesterol in proportions selected to approximate the major lipid classes of native LDL (Figure S12A). GMO cubosomes were selected because they displayed the most pronounced structural response to native LDL. Despite the complete absence of apolipoproteins, the lipid mimics induced concentration-dependent broadening and loss of the characteristic *Im*3*m* reflections. At the highest mimic/LCNP ratio investigated, the cubic reflections were replaced by a broad correlation maximum, indicating extensive disruption of the original cubic order. These results demonstrate that a simplified set of LDL-associated lipids is sufficient to induce LCNP remodeling and that apolipoproteins are not required to trigger structural reorganization in this model system.

We further investigated cholesterol individually because unesterified cholesterol is located within the LDL surface monolayer,^57^ making it readily available for molecular exchange with lipid membranes, and is a potent modulator of monoolein membrane packing and cubic-phase organization.^58,59^ At low cholesterol content, the *Im*3*m* reflections shifted toward lower *q* values, consistent with expansion of the cubic lattice, whereas higher cholesterol contents induced progressive loss of long-range order (Figure S12B). Thus, even a single exogenous lipid species can substantially reorganize the GMO matrix when incorporated at a sufficient concentration.

These reductionist experiments demonstrate that lipid components alone (from a simple selected mixture to an individual structurally active lipid) are sufficient to induce extensive LCNP remodeling. In the next section, we directly probe the insertion of LDL-derived lipid material into an LCNP-derived membrane using neutron reflectometry.

### Mechanism of Lipoprotein Corona-Induced Remodeling

To probe the molecular origin of the structural remodeling observed in LCNPs, we employed NR combined with an isotopic contrast-variation strategy. Unlike SAXS, which provides bulk-averaged structural information, NR yields subnanometer-resolved compositional profiles perpendicular to the interface, enabling high-resolution investigation of interfacial processes such as lipoprotein adsorption, lipid exchange, and membrane remodeling.^60,61^

To this purpose, we used solid-supported nanometric lipid films (“lipid carpets”) that replicate the outer structure of LCNPs, following a recently developed approach.^62,63^ These films are prepared by spin-coating of organic lipid solutions onto silicon substrates, followed by hydration (see Methods section). The process leads to highly ordered nonlamellar lipid carpets with nanometric thickness that faithfully reproduce the internal membrane organization of LCNPs (Figure 5A).

**Figure 5:**
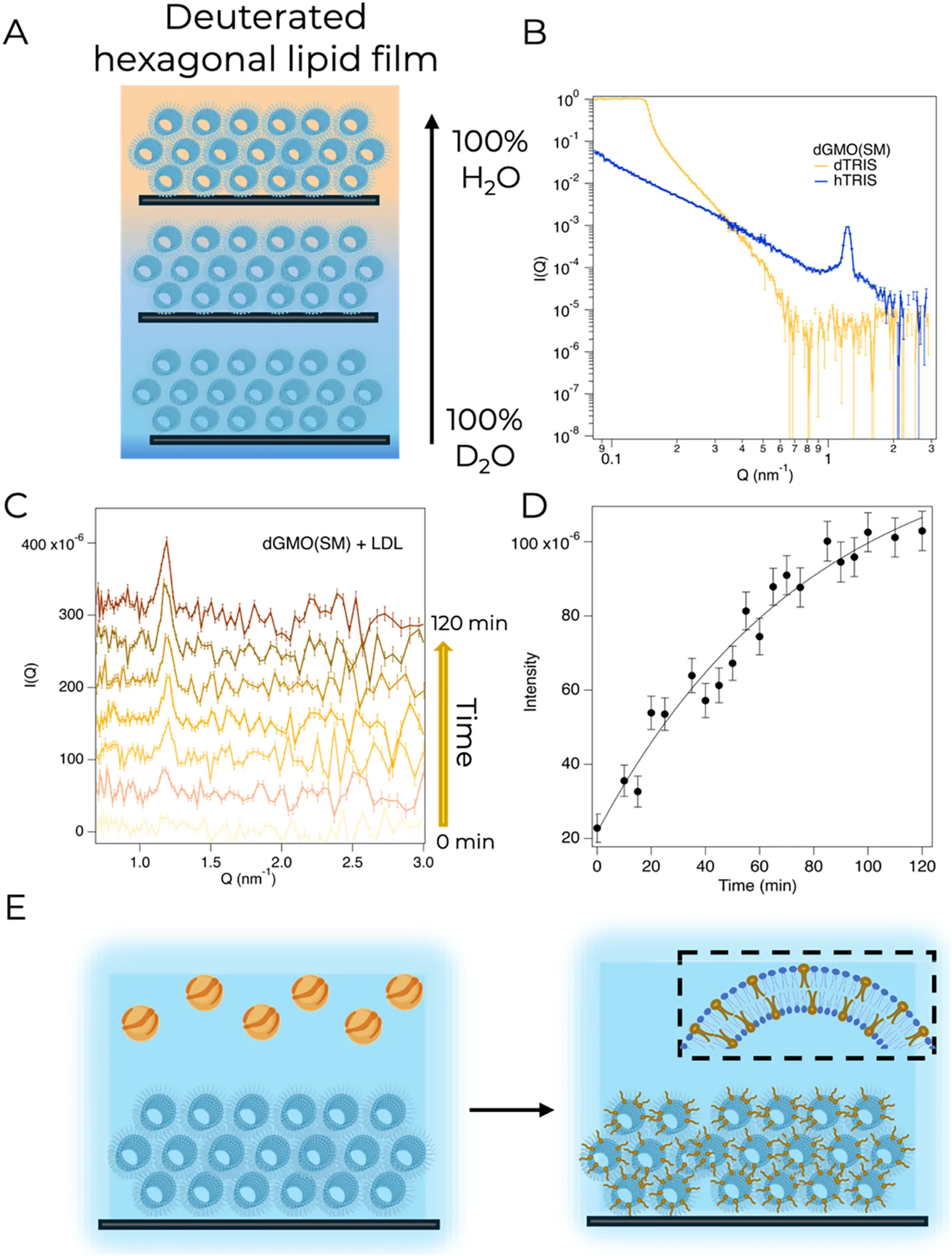
(A) Schematic representation of contrast conditions for dGMO­(SM) hexagonal-phase carpets obtained by varying H_2_O/D_2_O ratios. (B) NR profiles of dGMO­(SM) hexagonal lipid carpets measured in hTris (blue) and dTris (yellow). (C) Time evolution of the dGMO­(SM) NR profile following 1 mg mL^–1^ LDL injection in dTris. The NR profiles are vertically offset along the *y*-axis for clarity. (D) Time evolution of the Bragg peak intensity at *Q* = 1.2 nm^–1^ of the dGMO­(SM) carpet during 2 h of measurement. (E) Schematic illustration of hydrogenated components from LDL inserting into the hexagonal architecture of the GMO­(SM) carpet. Figure partially created in BioRender.

By combining these platforms with selective lipid deuteration, NR enables the simultaneous investigation of membrane restructuring and lipoprotein component insertion. Specifically, deuteration of the carpet lipids allows selective detection of structural changes within the LCNP membrane in hydrogenated aqueous media. Under these contrast conditions, lipoprotein-derived lipid components are nearly contrast-matched and therefore only weakly visible, so the measured signal primarily reflects structural rearrangements within the LCNP membrane (Figure 5A). Conversely, in deuterated media, lipoprotein lipids remain visible, enabling direct probing of lipid insertion from lipoproteins into the deuterated LCNP matrix.

To investigate these processes, we employed lipid carpets composed of deuterated oleoyl-chain GMO (dGMO; SLD = 5.5 × 10^–6^ Å^–2^) and SM-102 (90/10 mol %), reproducing the composition of GMO­(SM) LCNPs. This system was selected for its enhanced structural stability upon lipoprotein exposure, even at the relatively high concentrations required for NR measurements, thereby providing a robust platform to resolve interfacial exchange processes.

The NR profile of bare dGMO­(SM) carpets in 10 mM hydrogenated Tris buffer (hTris) (Figure 5B) exhibits a pronounced Bragg peak at *Q* = 1.2 nm^–1^, corresponding to the first-order reflection of the inverse hexagonal (H_II_) phase observed in GMO­(SM) LCNPs (Figure 1B). This confirms that the lipid carpet preserves the ordered internal structure observed at nanoparticle level. Upon exchange to deuterated Tris buffer (dTris), the Bragg peak disappears, indicating successful contrast matching of the deuterated lipid matrix: under these conditions, the lipid carpet does not contribute to the neutron scattering signal.

We then investigated lipoprotein interactions under contrast-matched conditions (dTris) to selectively probe lipoprotein binding and lipid transfer. A dispersion of hydrogenated LDL (1 mg mL^–1^) in dTris was injected into the NR cell, and interaction kinetics were monitored in real time using time-resolved NR over a reduced Q-range (see Methods section). Following LDL exposure, the Bragg peak, initially absent under contrast-matched conditions, progressively re-emerges and increases in intensity over time (Figure 5C). Importantly, the peak maximum remains centered at *Q* = 1.2 nm^–1^, matching the first-order reflection of the H_II_ phase observed in hydrogenated medium. The signal reaches a plateau after about 1 h, consistent with a dynamic yet finite exchange process (Figure 5D).

The recovery of the Bragg reflection associated with the inverse hexagonal phase, initially invisible under contrast-matched conditions, demonstrates that hydrogenated lipid species from LDL insert into the deuterated lipid matrix rather than remaining as intact lipoproteins adsorbed at the interface (Figure 5E). Their incorporation restores neutron scattering contrast in dTris, thereby recovering a Bragg signal that was initially detectable only in hTris.

These findings provide direct and unambiguous evidence that exogenous LDL-derived components undergo effective molecular exchange and structural integration into the LCNP-derived membrane. Such biomolecular exchange occurs even under conditions where only modest nanoparticle remodeling is detected, as in the case of GMO­(SM) LCNPs. More broadly, NR results validate molecular transfer as a central mechanism underlying the structural remodeling observed in dispersion-phase LCNPs and provide a mechanistic foundation for understanding how circulating lipoproteins can alter the internal architecture and, ultimately, the functional performance of lipid-based nanocarriers in biological environments.

### LCNP Remodeling in Biologically Relevant Environments

To assess whether lipoprotein-induced remodeling persists under physiologically relevant conditions, we investigated LCNP behavior in multicomponent lipoprotein environments. GMO cubosomes and GMO­(SM) hexosomes were incubated with a mixed HDL/LDL/VLDL formulation (lipoprotein/LCNP = 5 wt %), with relative lipoprotein proportions chosen to approximate plasma lipoprotein abundance in healthy individuals (see Methods section). The structural evolution and remodeling of LCNPs were monitored in real time by synchrotron SAXS in order to determine the time scales relevant to their remodeling in biological environments.

As shown in Figure 6A,B, both LCNP systems exhibit rapid structural changes, detectable at the earliest accessible time point (∼60 s), indicating that lipoprotein-induced remodeling essentially initiates immediately upon exposure. Despite this rapid onset, the two nanostructures respond differently. In analogy with the behavior observed with individual lipoproteins, GMO­(SM) hexosomes largely preserve their inverse hexagonal organization, although a progressive shift of the primary Bragg reflection toward higher *Q* values indicates lattice contraction. In contrast, GMO cubosomes exhibit a dramatically enhanced sensitivity to the mixed lipoprotein environment. Within 2 h of incubation, all Bragg reflections associated with the bicontinuous cubic phase disappear (Figure 6A), indicating complete loss of cubic internal order. At the same time, a broad, low-intensity scattering peak emerges at higher *Q*, consistent with the formation of a largely disordered lipid assembly, possibly accompanied by a partially organized structure that is nevertheless distinct from the original cubic phase.

**Figure 6:**
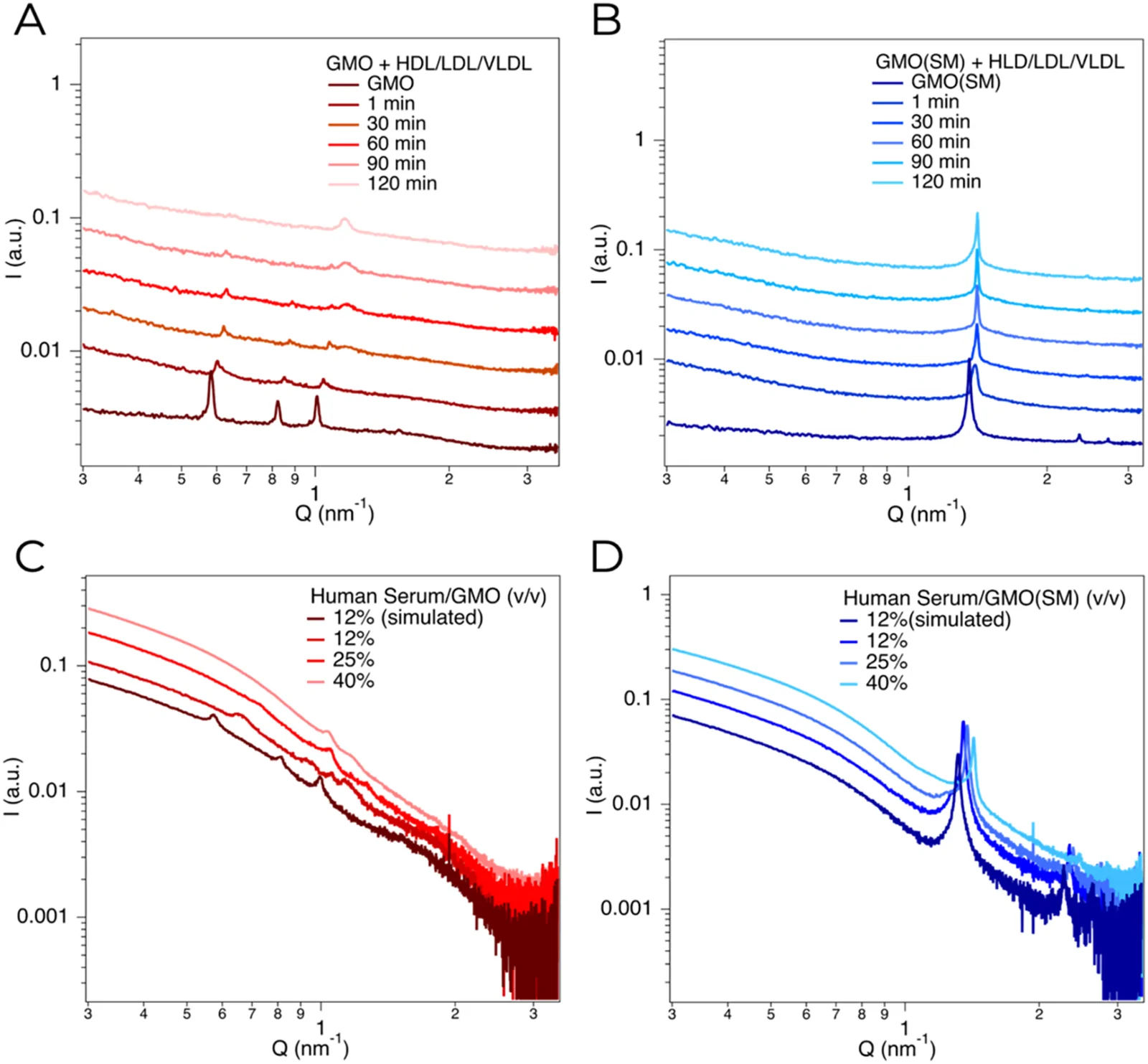
Time evolution of SAXS profiles of (A) GMO and (B) GMO­(SM) LCNPs interacting with HDL/LDL/VLDL mixtures (lipoproteins/LCNPs, 5 wt %) mimicking lipoprotein ratio in plasma from healthy donors. SAXS profiles of (C) GMO and (D) GMO­(SM) LCNPs incubated for 2 h with human serum at different volume ratios. The SAXS profiles are vertically offset along the *y*-axis for clarity.

These findings demonstrate that exposure to mixed lipoprotein systems exerts an even more pronounced destabilizing effect on LCNP internal structure than individual lipoprotein classes alone, underscoring the critical importance of investigating nanoparticle–corona interactions under physiologically realistic, multicomponent conditions.

We next extended the investigation of LCNP remodeling to a more complex biological environment by incubating LCNPs with human serum. In contrast with defined lipoprotein mixtures, human serum contains a wide variety of additional constituents, including abundant soluble proteins and other biomolecular species,^64^ which could in principle compete with lipoproteins for nanoparticle binding or otherwise modulate the remodeling process. As a result, serum components might partially shield LCNPs from lipoprotein interactions, thereby limiting or altering their structural reorganization.

To test this, LCNPs were incubated with increasing volumes of human serum, and their structural remodeling after 2 h was examined by synchrotron SAXS.

In contrast to defined lipoprotein mixtures, serum exhibits a strong and broad scattering background in the Q-range relevant for LCNP structural features, primarily arising from abundant proteins such as human serum albumin (HSA) (Figure S13).^65^ This background substantially lowers the apparent contrast of LCNP Bragg peaks, complicating the discrimination between peak attenuation due simply to background scattering and changes genuinely induced by LCNP–serum interactions.

To account for this effect, the experimental SAXS profiles of LCNPs in human serum were compared with simulated curves obtained by linearly combining the scattering contributions of LCNPs and serum. These simulated profiles represent the expected signal in the absence of any interaction between the two systems (Figures 6C,6D, S14 and S15).

Despite the reduced contrast for LCNP-related Bragg features, clear structural trends are observed. For GMO cubosomes, increasing serum concentration induces a progressive shift of the *Im*3*m* reflections toward higher *q* values, followed by their complete disappearance and replacement by a weak, broad feature at *Q* ≈ 1.2 nm^–1^. GMO­(SM) hexosomes exhibit a similar but less pronounced response, with hexagonal reflections shifting toward higher Q values while remaining mostly preserved, indicating greater structural resilience.

To assess whether an abundant soluble serum protein could independently induce a comparable structural response, both LCNP formulations were incubated for 2 h with purified human serum albumin (HSA, the most abundant serum proteins) at concentrations ranging from 10 to 50 mg mL^–1^ (Figure S16). Because HSA contributes substantial scattering in the same *Q*-range as the LCNP structural features, the experimental profiles obtained at 50 mg mL^–1^ HSA were compared with simulated noninteracting profiles generated by linear combination of the separately measured LCNP and HSA scattering curves. No systematic shifts, broadening or variation in the intensity of the LCNP Bragg reflections were observed relative to the corresponding noninteracting profiles. HSA alone therefore did not induce detectable remodeling of either internal phase under the conditions investigated.

The structural evolution observed in serum closely resembles that induced by defined lipoprotein mixtures. Together with the absence of detectable remodeling upon exposure to HSA alone, this similarity supports lipoproteins as major contributors to the serum-induced structural response of the LCNPs, consistent with a mechanism based on lipid exchange and compositional remodeling, while not excluding possible contributions from other serum constituents. Taken together, these results demonstrate that a remodeling pattern consistent with that induced by lipoproteins persists in the complex environment of human serum. This provides direct evidence that corona formation in biological fluids can profoundly reshape nanoparticle internal architecture. Importantly, the strong LCNP phase dependence of this response highlights that the internal nanostructure is a key determinant of LNP adaptation under physiologically relevant conditions.

### Impact of Lipoprotein-Induced Remodeling on Cell Uptake

As a proof of concept to link corona-driven structural transformations to biological performance, we investigated the cellular uptake of LCNPs using the T24 cell line.

Cellular uptake was evaluated by confocal laser scanning microscopy using β-bodipy-labeled LCNPs. GMO cubosomes and GMO­(SM) hexosomes were either used as-prepared or preincubated with LDL (2 h, LDL/LCNP = 5 wt %) prior to cell exposure. A plasma membrane marker was used to delineate cell boundaries and distinguish intracellular fluorescence. Uptake experiments were performed in serum-free medium (DMEM) to eliminate interference from endogenous proteins and lipoproteins,^66^ thereby isolating the effect of the preformed lipoprotein corona (see Materials and Methods). The negligible impact of DMEM on the structure of both LCNP formulations is shown in Figure S17.

In the absence of LDL preincubation, both LCNP formulations showed measurable cellular internalization after 4 h of incubation (Figure 7A,B). GMO­(SM) hexosomes exhibited moderately higher uptake compared to GMO cubosomes, likely due to their slightly positive surface potential, resulting from the incorporation of the ionizable lipid SM-102, driving favorable electrostatic interactions with negatively charged cell membranes.^67^

**Figure 7:**
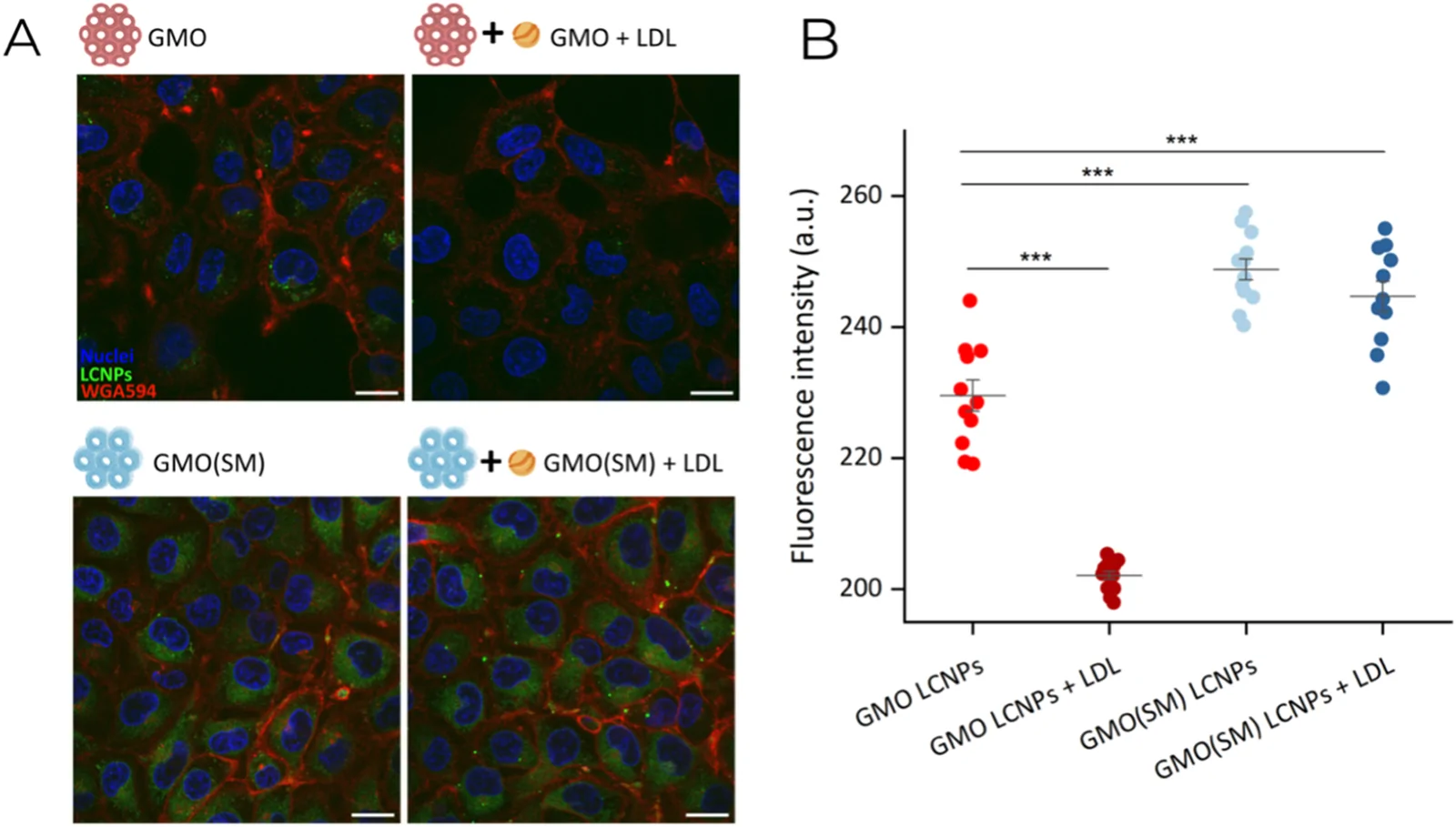
Cellular uptake of LCNPs. (A) Representative confocal images of T24 cells incubated for 4 h with GMO, GMO + LDL, GMO­(SM) and GMO­(SM) + LDL LCNPs labeled with β-Bodipy and plasma membrane labeled with WGA Alexa Fluor 594. Green, LCNP; red, plasma membrane; blue, nuclei. Scale bar: 20 μm. (B) Quantification of cellular uptake of LCNPs in T24 cells. Fluorescence intensity (a.u.) measured by confocal microscopy after 4 h incubation. More than 10 images were analyzed from 3 independent experiments. Error bars, SE. Asterisks indicate significant differences determined by one-way ANOVA followed by Bonferroni and Tukey multiple comparison post hoc tests (*** *p* < 0.001). Figure partially created in BioRender.

Strikingly, preincubation with LDL produced markedly different outcomes for the two nanostructures. GMO cubosomes exhibited a significant reduction in cellular uptake (≈10%), as evidenced by decreased intracellular fluorescence intensity and quantitative image analysis. In contrast, LDL exposure did not produce a statistically significant change in the uptake of GMO­(SM) hexosomes.

These divergent biological responses cannot be explained solely by differences in lipoprotein adsorption, as f-NTA measurements demonstrated comparable levels of LCNPs associated with LDL for both formulations (Figure 2A,B). Instead, they directly reflect the distinct structural responses of the two LCNP architectures upon lipoprotein interaction (Figure 3). As shown by SAXS, cryo-EM, and DLS, GMO cubosomes undergo extensive internal restructuring and size reduction following LDL exposure, leading to loss of long-range order and formation of disordered, small lipid assemblies. In contrast, GMO­(SM) hexosomes largely preserve their internal organization, with only moderate lattice contraction and limited morphological perturbation.

The reduction in uptake observed for GMO cubosomes is therefore associated with the extensive structural remodeling measured by SAXS, cryo-EM, and DLS, including changes in particle size, internal membrane organization, and interfacial morphology. Conversely, the structural resilience of hexosomes preserves these features, maintaining their uptake efficiency despite LDL association.

### Mechanistic Hypothesis

Over the past decade, substantial efforts have been devoted to elucidating the formation and biological implications of the biomolecular corona on nanoparticles.^68^ This body of work has focused predominantly on protein adsorption, corona composition and isolation, and how these factors influence key biological processes such as cellular uptake and biodistribution.^69^ A growing number of studies have demonstrated that specific corona constituents, particularly apolipoproteins, play an active role in mediating nanoparticle–cell interactions by engaging endogenous transport pathways and directing organ-specific accumulation.^12,13^ In parallel, considerable progress has been made in isolating and characterizing the protein corona using advanced proteomic techniques, alongside efforts to rationally engineer nanoparticle surfaces to modulate corona composition.^70–72^

Collectively, the current understanding of biomolecular corona formation is largely based on a surface-dominated framework, in which adsorbed biomolecules modulate nanoparticle identity without significantly affecting internal structure, a perspective largely inherited from studies on rigid inorganic nanoparticles. While appropriate for rigid nanomaterials, this view is less established for soft lipid-based systems, where dynamic molecular exchange may couple interfacial processes to internal organization.

Recent studies have begun to hint at this possibility, suggesting that some protein corona components can induce compositional rearrangements or surface restructuring.^11,21^ In particular, Sebastiani et al. showed that isolated ApoE can induce lipid redistribution within MC3-based mRNA-LNPs. Although this study investigated a single apolipoprotein rather than intact native lipoproteins, it supports the possibility that related remodeling processes may also occur in clinically relevant LNP formulations. While these studies provide important early indications that corona interactions may extend beyond simple adsorption, they do not establish whether whole lipoproteins induce substantial or large-scale restructuring of LNPs. Importantly, the impact of the protein corona on structurally sensitive systems, such as liquid crystalline lipid nanoparticles (LCNPs), where internal architecture plays an even more key role in determining function and that are increasingly considered as next-generation lipid carriers, remains overlooked.

In this work, we demonstrate that plasma lipoproteins actively participate in LNP remodeling through lipid exchange with the nanocarrier membrane. Using a combination of SAXS, cryo-EM, fluorescence nanoparticle tracking, and neutron reflectometry, we show that this exchange is accompanied by phase-dependent structural reorganization of lipid nanoparticles across multiple length scales. Importantly, the extent of remodeling depends strongly on nanoparticle architecture. Bicontinuous cubic LCNPs undergo pronounced disruption, including loss of long-range order and size reduction, whereas inverse hexagonal systems retain their mesophase with only moderate lattice contraction. These differences highlight that internal phase structure is a key determinant of nanoparticle resilience in biological environments.

Mechanistically, our results support a model in which corona formation is coupled to lipid exchange between lipoproteins and LNP membranes, leading to compositional changes that alter curvature stress and drive structural rearrangement. Neutron reflectometry provides direct evidence of this lipid insertion from lipoproteins into model LNP membranes, confirming molecular transfer as the origin of the observed effects. This mechanism is schematically illustrated in Figure 8.

**Figure 8:**
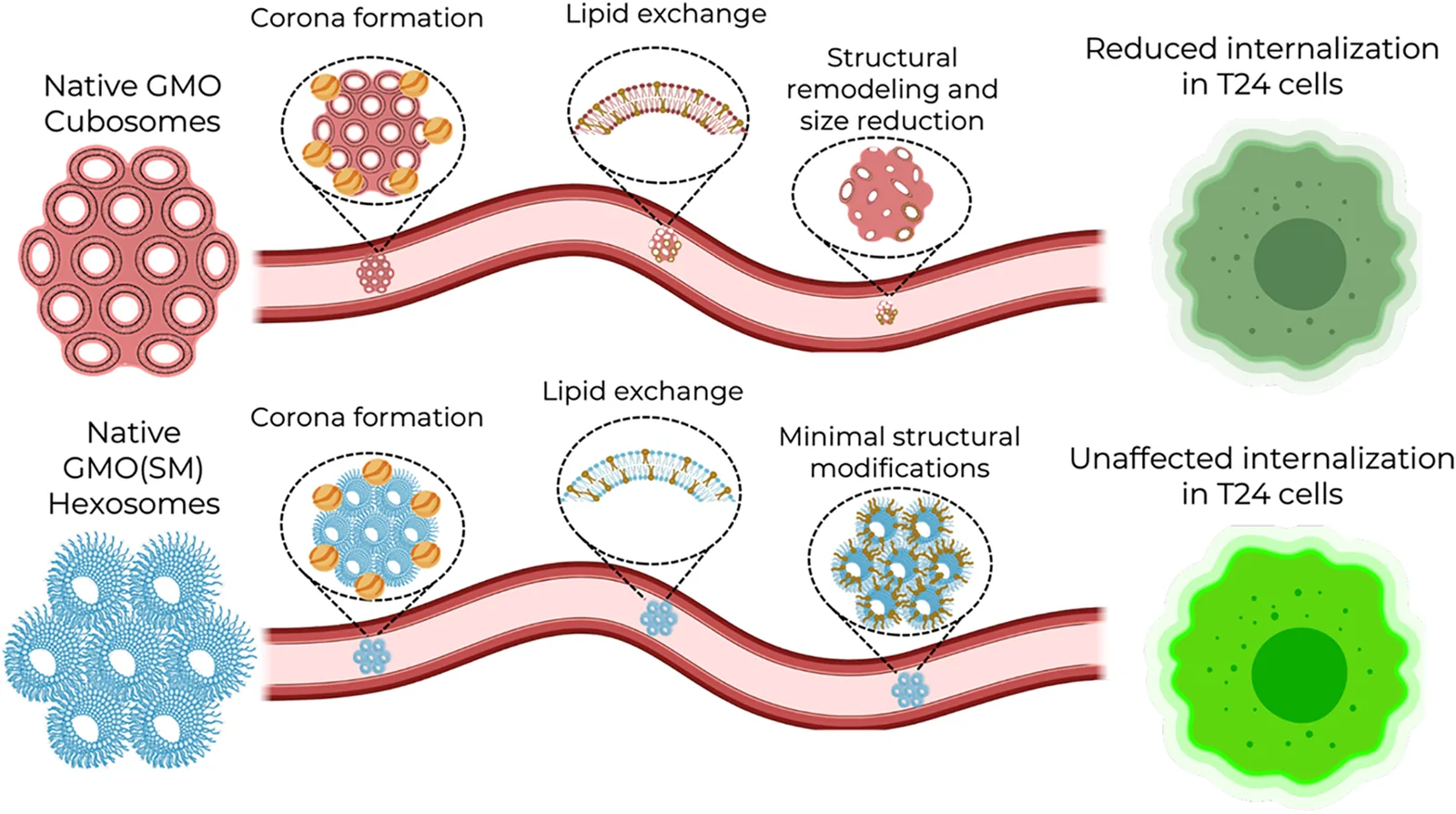
Schematic representation of the lipoprotein-driven structural remodeling of LCNPs through coupled corona formation and lipid exchange. Figure partially created in BioRender.

Together, these results indicate that lipoproteins do not persist as intact particles at the LCNP interface, but instead exchange lipid material with the membrane, consistent with recent evidence of lipoprotein–LNP fusion.^73^ This raises a broader conceptual point: the classical notion of a biomolecular corona, developed for rigid, inorganic nanoparticles and implying a shell of intact adsorbed biomolecules on an otherwise static surface, may not adequately capture interfacial phenomena in soft, structurally dynamic lipid nanocarriers, where association and compositional exchange are mechanistically coupled. This distinction is only beginning to emerge in the literature, and we see the present work, together with very recent, independent evidence,^21,73^ as an early step toward a terminology and conceptual framework better suited to describe corona-like phenomena in soft nanomedicine.

Because lipoprotein association and molecular exchange occur at the LCNP interface, the surface stabilizer may also influence these processes. In the present formulations, Pluronic F127 provides steric stabilization through its hydrated PEO chains. Although F127 did not prevent lipoprotein association or structural remodeling under the conditions investigated, its interfacial layer may modulate the extent and kinetics of these processes through steric shielding. Previous studies have shown that the influence of F127 on protein adsorption and corona formation is system-dependent, ranging from reduced adsorption of selected plasma proteins^74,75^ to limited shielding of the nanoparticle surface.^76^ The specific role of F127 in LCNP–lipoprotein interactions therefore remains an important topic for future investigation.

Finally, the observed structural remodeling correlates with changes in cellular uptake, suggesting that internal architecture modulates biological performance beyond surface effects alone. Since nanoparticle internalization is strongly cell-type dependent, future studies should determine whether the structure–uptake relationship observed here is maintained in physiologically relevant cell types, including macrophages, hepatocytes, and muscle cells.

We note also that the changes in cellular internalization observed here may not directly translate into corresponding changes in endosomal escape or functional RNA delivery.^14^ Future studies should therefore directly assess how lipoprotein-induced structural remodeling affects cargo retention, intracellular trafficking, endosomal escape, and functional delivery.

Moreover, the present study was performed under static conditions, whereas nanoparticles in vivo are exposed to continuous flow and shear stress. Dynamic exposure conditions may alter the kinetics and composition of the biomolecular corona^77–79^ and, consequently, influence lipoprotein exchange, structural remodeling, and the resulting biological responses. These effects should be investigated in future studies performed under physiologically relevant flow conditions.

Although the biological consequences of remodeling were demonstrated in a simplified cellular system and under static conditions, these findings indicate that resistance to premature and uncontrolled structural remodeling upon exposure to serum and lipoproteins, prior to and during cellular uptake, should represent an additional design parameter for lipid-based nanocarriers.

By establishing the occurrence, phase dependence, and molecular basis of lipoprotein-induced remodeling in structurally diagnostic model systems, the present study provides a mechanistic foundation for extending this investigation to more compositionally complex and clinically relevant formulations. Future studies should determine whether intact native lipoproteins induce comparable remodeling in clinically relevant SM-102- and MC3-based LNPs, as well as in PEGylated liposomal formulations such as Doxil, using complementary structural approaches where symmetry-specific Bragg reflections are absent or weak.

## Conclusion

In summary, we show that plasma lipoproteins can directly exchange lipids with lipid nanoparticles, driving phase-dependent remodeling of their internal structure. Remodeling was observed with each of the three lipoprotein classes tested and remained evident in defined lipoprotein mixtures and human serum, highlighting its relevance under physiologically realistic conditions. Consistent with a major contribution from lipoproteins, HSA alone did not induce detectable structural remodeling under the conditions investigated. Our results establish that, in soft lipid nanoparticles, the biological identity commonly described as a biomolecular corona is not necessarily established through surface adsorption alone. Instead, lipoprotein association can involve molecular exchange and structural integration of lipoprotein-derived material, thereby reshaping the nanoparticle internal architecture. Importantly, the extent of this remodeling is strongly associated with the original internal phase architecture of the lipid nanocarrier.

These findings identify internal structural organization as a critical determinant of nanoparticle stability in biological environments and suggest that resistance to lipid exchange should be considered alongside conventional design parameters such as size and surface chemistry. More broadly, this work provides a framework for understanding how lipid-rich biological components actively participate in the self-organization of soft nanomaterials in vivo.

## Methods

### Materials

Glyceryl Monooleate (GMO) was sourced from Croda (Cithrol GMO HP, minimum glyceryl monooleate content of 92%). Glyceryl monooleate-d_32_ (dGMO) was synthesized according to literature procedure with slight modification^80^ (see SI for the details). The ionizable lipid 8-[(2-hydroxyethyl)­[6-oxo-6-(undecyloxy)­hexyl]­amino]-octanoic acid, 1-octylnonyl ester (SM-102) (≥99.9%) was provided by CABRU s.a.s. (Arcore, Italy). The synthetic triblock copolimer Pluronic F-127 (≥99.9%) was purchased from Merck. β-Bodipy FL C12 HCP (≥99%) and Alexa fluor 647 NHS were purchased from Invitrogen (Thermo Fisher Scientific). Ethanol absolute (≥99.9%) was purchased from Carlo Erba (Milan, Italy). Trizma base (Tris­(hydroxymethyl)­aminomethane, ≥99.9%) and sterile-filtered human serum were purchased from Merck. Disposable and cell culture plasticware were purchased from Sarstedt Ag & Co. KG, (Nümbrecht, Germany). HDL, LDL, and VLDL from human plasma were purchased from Medix Biochemica (Espoo, Finland). Phosphate Buffered Saline (PBS) was purchased from Lonza (Basel, Switzerland). 0.45 μm Primo PES syringe filters used to filter PBS were purchased from Euroclone S.p.A. (Milan, Italy). All products for cell culture were purchased from Thermo Fisher Scientific (Waltham, MA, USA) as well as the products used in confocal microscopy experiments. Cells were obtained from the American Type Culture Collection (Manassas, VA, USA). 1-Palmitoyl-2-oleoyl-*sn*-glycero-3-phosphocholine (POPC) (≥99.9%), triolein (≥99.9%), cholesteryl oleate (≥99.9%), and cholesterol (≥99.9%) were purchased from Merk. Human serum albumin (HSA; ≥95% (agarose gel electrophoresis)) was purchased from Merk.

### Lipoproteins Labeling and Purification

Lipoproteins were fluorescently labeled with Alexa Fuor 647 NHS via EDC/NHS chemistry, following the protocol reported previously.^52^

After incubation with Alexa Fluor 647, particles were purified from excess probe through SEC.^53^ Labeled particles were loaded on top of a Tricorn 10/300 column (Cytiva, US) packed with Sepharose CL-4B resin (Sigma-Aldrich, Germany). The purification was controlled through an AKTA Pure FPLC system (Cytiva, US), equipped with a fluorescence detector to track the elution labeled particles. Fluorescence at λ_exc_ = 633 nm and λ_em_ = 700 nm were collected and used to track lipoproteins. Flow rate was set to 0.4 mL/min, using degassed sterile PBS as eluent buffer. Fractions eluting between 7 and 8 mL were collected, combined and further analyzed.

Protein quantification in the samples was performed using a Pierce Micro BCA Protein Assay kit (Thermo Fisher Scientific, MA, US), following the “Test Tube Procedure” indicated on the manufacturer’s protocol (see Figure S1).

### LCNPs Preparation

LCNPs were produced using a custom-designed 3D-printed polypropylene microfluidic device featuring two separate inlet channels for the organic and aqueous phases.^81^ The two streams merge at a T-junction and flow into a single main channel incorporating a zigzag bas-relief geometry to promote enhanced mixing under laminar flow conditions. The microchannels have a square cross-section with a width of 1 mm, while the patterned bas-relief structure has a height of 500 μm. The total length of the main channel is 60 mm.

Both phases were injected into the device using a precision syringe pump system. The organic phase consisted of a 50 mg/mL lipid solution in ethanol, containing either 100 mol % GMO to produce cubosomes or GMO and SM-102 at a 90:10 molar ratio to produce hexosomes. The aqueous phase consisted of 10 mM Tris buffer (pH 7.4) containing Pluronic F127, a nonionic triblock copolymer composed of poly­(ethylene oxide) (PEO) and poly­(propylene oxide) (PPO) arranged in a PEO–PPO–PEO sequence, at 10 wt % relative to the total lipid content. Pluronic F127 was used as a steric stabilizer to prevent aggregation and obtain colloidally stable dispersions of cubosomes and hexosomes.

During nanoparticle formation, the aqueous-to-organic flow rate ratio was maintained at 3:1, with a total flow rate of 20 mL/min, yielding a final ethanol concentration of approximately 25% (v/v) at the device outlet (flow conditions were selected based on preliminary optimization experiments aimed at minimizing particle size and polydispersity).

Following microfluidic assembly, ethanol was removed by dialysis against 10 mM Tris for 12 h using a cellulose membrane with a molecular weight cutoff of 14 kDa. Typically, 2 mL of the nanoparticle suspension was dialyzed against approximately 250 mL of Tris, which was refreshed at least once during the process.

### Dynamic Light Scattering

The hydrodynamic diameter and polydispersity index (PDI) of GMO and GMO­(SM) LCNPs and they mixture with LDL were determined using dynamic light scattering (DLS) with a Brookhaven Instrument 90 Plus (Brookhaven, Holtsville, NY). Each measurement was the average of 3 repetitions of 1 min each. The autocorrelations functions were analyzed using the cumulant algorithm stopped at the second order and the CONTIN algorithm. Samples were diluted with Milli-Q water to 0.1 mg mL^–1^ lipid concentration. DLS measurement was collected in triplicate at 90°.

### Small Angle X-ray Scattering

Small-angle X-ray scattering (SAXS) experiments were performed at three different synchrotron beamlines. The data sets shown in Figures 1 and 5 were acquired at the SAXS beamline of the Elettra synchrotron (Trieste, Italy), which operates at an electron energy of 2.0 GeV. Measurements were carried out using an X-ray beam with an energy of 8 keV (λ = 0.154 nm), and scattering profiles were recorded with a Pilatus 3 1 M detector. Samples were loaded into borosilicate glass capillaries with a wall thickness of 1.5 mm (WJM Glas Müller GmbH, Berlin, Germany).

The data presented in Figures 3 and 6A,B were collected at beamline ID02 of the European Synchrotron Radiation Facility (ESRF, Grenoble, France). A monochromatic X-ray beam with a wavelength of 1.00 Å (12.23 keV) and a sample-to-detector distance of 2 m were used, providing access to a scattering vector range of 0.35–4.218 nm^–1^. Two-dimensional scattering patterns were recorded using a Rayonix MX-170HS detector with a pixel size of 44 × 44 μm^2^. Samples were measured in 1.5 mm thick glass capillaries.

Additional SAXS measurements (Figure 6C,D) were performed at beamline B21 of the Diamond Light Source (UK). Samples were loaded into a 96-well plate using an EMBL BioSAXS robot and automatically transferred into a quartz capillary with an inner diameter of 1.8 mm positioned in the X-ray beam. This beamline was operated with a fixed camera length of 3.9 m and a wavelength of 1.00 Å, and data were collected using a Pilatus 2 M detector. Data reduction, including background subtraction and radial averaging, was carried out with the Scatter software.

The lattice parameters of the cubic and inverse hexagonal (*d*) phases were determined from the positions of the Bragg peaks. For the cubic phase, *d*_*c*_ was calculated from each reflection (*q*_*hkl*_) according to

$$d=\frac{2\pi }{q_{\mathrm{hkl}}}\sqrt{h^{2}+k^{2}+l^{2}}$$

where *h*, *k*, and *l*are the Miller indices. For the inverse hexagonal phase, the lattice parameter d was obtained from the peak positions (*q*_*hk*_) using

$$d=\frac{4\pi }{\sqrt{3}q_{\mathrm{hk}}}\sqrt{h^{2}+\mathrm{hk}+k^{2}}$$

where *h* and *k* are the Miller indices of the two-dimensional hexagonal lattice.

The radii of the aqueous channels were then estimated using geometrical models. For the cubic phase, the water channel radius (*r*_w_) was calculated based on the curvature of triply periodic minimal surfaces

$$r_{w}=\left(d\right)-l$$

where *A*_0_ is the surface area per unit volume, χ is the Euler–Poincaré characteristic, and *l* is the hydrophobic chain length, assumed to be 18 Å. For the inverse hexagonal phase, the water channel radius (*r*_w_) was estimated as

$$r_{w}=\frac{d}{2}-l$$

For SAXS measurements of LCNP–lipoprotein samples, LCNP dispersions (12.5 mg mL^–1^, 10 mM Tris buffer, pH 7.5) were incubated with lipoprotein dispersions to obtain a final lipoprotein/LCNP weight ratio of 5 wt %, unless otherwise specified. For concentration-dependent SAXS experiments, the LCNP concentration was kept constant (12.5 mg mL^–1^), while lipoprotein stock dispersions of appropriate concentrations were used to achieve the desired final lipoprotein/LCNP weight ratios without diluting the LCNP dispersion.

For the experiments shown in Figure 6A,B, a mixture of lipoproteins was used while maintaining the same total lipoprotein/LCNP weight ratio (5%). The relative proportions of HDL, LDL, and VLDL were selected to approximate their physiological abundance in human serum.^82^ Accordingly, the mixture consisted of ∼31 wt % HDL, 63 wt % LDL, and 6 wt % VLDL.

For the experiments with human serum (Figure 6C,D), 40 μL of LCNPs (12.5 mg mL^–1^) has been incubated with increasing volumes of serum (7 μL, 13 μL, and 25 μL, reaching final volume ratios of 12%, 25%, and 40%).

### Neutron Reflectometry

Neutron reflectometry (NR) experiments were performed on the FIGARO (Fluid Interfaces Grazing Angles ReflectOmeter) time-of-flight reflectometer at the Institut Laue–Langevin (ILL, Grenoble, France), using data from the ILL data set reported in ref 83 FIGARO is a high-flux neutron reflectometer operating with a vertical scattering geometry and optimized for the investigation of soft-matter interfaces, including air/liquid and solid/liquid systems. Measurements were conducted using a wavelength band of 20 Å, providing access to a momentum transfer range (*q*) of approximately 0.009–0.3 Å^–1^ through the use of two incident angles (0.7° and 3.0°). The reflected neutron intensity was recorded using a two-dimensional position-sensitive detector, enabling determination of interfacial structure with subnanometer resolution. The instrument was configured to provide a Δ*Q*/*Q* resolution of approximately 5% with the high neutron flux of the ILL, facilitating rapid characterization of lipid–lipoprotein interfacial organization. Data reduction and normalization were performed using standard ILL reflectometry procedures.

Briefly, lipid films were prepared by spin-coating (Delta 6 RC TT spin coater, SÜSS MicroTec Lithography GmbH) LCNP lipid solutions in chloroform (10 mg/mL of dGMO and SM-102, 90/10%mol) onto polished silicon substrates (horizontal orientation, block size 50 × 80 × 15 mm^3^), followed by solvent evaporation (calculated scattering length density: 4.9 × 10^–6^ Å^–2^ for dGMO/SM-102). The resulting dry films were subsequently hydrated with Tris buffer (10 mM, pH 7.5) to promote formation of supported lipid nanoparticle layers and mounted in solid–liquid NR cells.

Each sample was characterized using NR under two solvent contrasts: (i) hydrogenated Tris buffer (100 vol % H_2_O), to probe the overall structure of the deuterated LCNP layer; (ii) deuterated Tris buffer (100 vol % D_2_O), contrast-matched to the LCNP matrix to enhance sensitivity to lipid insertion into the LCNP matrix from lipoproteins. Following LCNP matrix measurements, LDL solution (1 mg mL^–1^) prepared in LCNP contrast-matched buffer were injected into the NR cell. Time-resolved NR measurements were then performed at a fixed incident angle of 1.2° over a period of 2 h, with an acquisition time of 60 s per spectrum, to monitor lipoprotein interaction and lipid exchange dynamics at the LCNP interface in real time.

### Nanoparticle Tracking Analysis

Colocalization between fluorescently labeled LCNPs (cubosomes or hexosomes) and lipoproteins were determined using a Particle Metrix ZetaView x30 QUATT instrument. Laser alignment and detector calibration were performed using TetraSpeck 100 nm polystyrene nanoparticle standards (Particle Metrix, Germany), diluted 1:1000 in filtered 10 mM Tris buffer pH 7.5, according to the manufacturer’s instructions.

LCNPs were used at a fixed concentration of 0.25 mg mL^–1^ and incubated with increasing amounts of lipoproteins (stock concentration 0.044 mg mL^–1^) to reach final lipoprotein/LCNP weight ratios ranging from 0 to 10 wt %. After 2 h of incubation, samples were diluted 100-fold to reach optimal particle concentrations and analyzed by f-NTA. For the experiments reported in Figure 2A, LCNPs (0.25 mg mL^–1^) were incubated with a lipoprotein mixture containing HDL, LDL, and VLDL in a 1:1:1 ratio, reaching a final lipoprotein/LCNP weight ratio of 5%.

1.2 mL of the solution was injected using a disposable 1.5 mL sterile plastic syringe avoiding the formation of air bubbles. The sensitivity for the scattering channel was set to 60. The sensitivity for the fluorescence channels was set to 85. Camera shutter was set to 200. Particle total scattering, fluorescence at λ_em_ = 520 nm and λ_em_ = 670 nm, as well as colocalization signals were recorded and analyzed using pEX integrated software or custom python scripts.

### Cryogenic Electron Microscopy

Cryo-EM experiments were carried out at the Cryo-Electron Microscopy Laboratory of ISASI-CNR (Naples, Italy). For grid preparation, 5 μL of each sample were deposited onto glow-discharged Lacey Formvar/Carbon grids 200 mesh (Quantifoil, Großlöbichau, Germany) and vitrified in liquid ethane using a Vitrobot Mark IV (Thermo Fisher Scientific, Waltham, MA, USA) operated at 4 °C and 100% relative humidity. Prior to vitrification, GMO and GMO­(SM) LCNPs were diluted to a final concentration of 1 mg/mL. To prepare GMO + LDL and GMO­(SM) + LDL samples, LCNPs were first incubated with LDL for 2 h and subsequently diluted to 1 mg/mL (LDL/LCNPs 5 wt %). Grids were blotted for 2 s after a 10 s wait time to facilitate particle distribution within the grid holes.

Data were collected using a Glacios transmission electron microscope (Thermo Fisher Scientific) operated at 200 kV with a 50 μm C2 aperture. Images were recorded in nanoprobe EFTEM mode at nominal magnifications of 39.000×, 79.000× and 130.000×, with a total electron dose of 20 e^–^/Å^2^ and defocus values ranging from −1.5 to −2 μm. A Falcon 4i direct electron detector was used in combination with a Selectris energy filter (Thermo Fisher Scientific) operated in zero-loss mode with a 10 eV slit. Parallel illumination was achieved by aligning the beam using diffraction on a carbon foil to minimize imaging artifacts. Astigmatism and coma were corrected with Sherpa software, and the energy filter was tuned to the zero-loss peak prior to each acquisition area. Automated data collection and image processing were performed using Smart EPU and Velox software (Thermo Fisher Scientific), respectively.

### Cell Uptake

T24 bladder carcinoma epithelial-like cells (American Type Culture Collection, Manassas, VA, USA) were cultured in Dulbecco’s Modified Eagle’s Medium (DMEM; Thermo Fisher Scientific, Waltham, MA, USA) supplemented with 10% fetal bovine serum (FBS) and 1% penicillin–streptomycin. T24 cells were selected as a simple and reproducible proof-of-concept model to examine the relationship between lipoprotein-induced structural remodeling and cellular uptake at the biophysical level. Cells were cultured at 37 °C in a humidified incubator with 5% CO_2_ and allowed to proliferate until reaching approximately 90% confluence.

### Confocal Microscopy Uptake Studies

T24 cells were plated on 18 mm glass coverslips in 12-well plates at 100.000 cells per well. 24 h after plating, cells were incubated with 0.1 mg mL^–1^ LCNPs labeled with β-Bodipy FL C12 HCP and LCNPs labeled with β-Bodipy FL C12 HCP previously mixed with LDL (for 2 h (LDL/LCNPs 5 wt %)) for 4h in serum-free medium (to prevent interactions between LCNPs and serum proteins). Cells were then incubated for an additional 1h in complete medium to remove any unbound dye. Subsequently, they were incubated with 5 μg/mL WGA Alexa Fluor 594 (ThermoFisher Scientific) for 20 min to specifically label the plasma membrane. Ten minutes before the end of this incubation time, cells were incubated with Hoechst 33342 (ThermoFisher Scientific) dye at a concentration of 10 μg/mL to stain the nuclei of the cells. After the incubation, cells were washed with PBS, mounted on a custom chamber for imaging and the medium was replaced with Leibovitz’s L-15 (ThermoFisher Scientific), a medium designed for supporting cell growth in the absence of CO_2_ equilibration.

The analysis was performed with a Nikon Eclipse TE300C2 LCSM (Nikon) equipped with a Nikon Plan Apo λ 100× 1.49 NA oil immersion objective and with Coherent CUBE (diode 405 nm), Melles Griot (Argon 488 nm) and Coherent Sapphire (Sapphire 561 nm) lasers. Emission filters for imaging were 452/45 nm, 514/30 nm and 595/60 nm. For each sample, optical sections at median planes of the cells were taken (1024 × 1024 pixels) using subsaturation settings and all of them were kept constant for each analysis (laser power, detector gain, and pinhole diameter). Images were processed and analyzed with the Fiji software and Origin (Pro) (“Version 2022b”, OriginLab Corporation). One-way ANOVA followed by Bonferroni and Tukey multiple comparison post hoc tests were used. The level of significance was set at *p < 0.05, **p < 0.01, and ***p < 0.001. A minimum of 3 independent experiments were performed, and more than 10 images per experiment were analyzed.

## Supplementary Material



## Notes

Additional details on lipoprotein purification and characterization, DLS and cryo-TEM characterization of LCNPs and lipoproteins, fluorescence NTA measurements, SAXS characterization of LCNP–lipoprotein interactions, control experiments with LDL-inspired lipid mimics and HSA, and the effect of cell culture medium on LCNP structure; additional structural and morphological characterization of GMO and GMO­(SM) LCNPs upon interaction with LDL; synthesis and NMR characterization of deuterated GMO (d-GMO); experimental methods and protocols (PDF)
