# Supplementary material for: Lipoprotein Corona Reshapes the Internal Structure of Lipid Nanoparticles
Source: ACS Nano. 2026 Sep 2;20(36):24915–33. doi: 10.1021/acsnano.6c09400 (PMC13585173; doi:10.1021/acsnano.6c09400)
Supplement: Supplementary file 1 [file nn6c09400_si_001.pdf]

Supporting Information

Lipoprotein Corona Reshapes the Internal Structure of Lipid Nanoparticles

Lucrezia Caselli[1,2], Marta Rojas-Rodríguez[3], Valentina Pacciani[1,2], Martino Calamai[3,4], Roberto Frigerio[5], Andrea Zendrini[5], Gennaro Sanità[6], Emanuela Esposito[6], Anna Leung[7], Hanna Wacklin-Knecht[7], Ben Humphreys[8], Paolo Arosio[5], Jacopo Cardellini[1,2]*, and Debora Berti[1,2]*

Affiliations:

[1]Department of Chemistry "Ugo Schiff", University of Florence, 50019 Sesto Fiorentino, Florence, Italy;

[2]CSGI, Center for Colloid and Surface Science, Sesto Fiorentino 50019 Florence, Italy

[3]European Laboratory for Non-Linear Spectroscopy, via Nello Carrara 1, 50019 Sesto Fiorentino, Italy;

[4]National Institute of Optics - National Research Council Via Nello Carrara 1 - I 50019 Sesto-Fiorentino (Firenze) – Italia;

[5]ETH Zürich, Department of Chemistry and Applied Biosciences, Institute for Chemical and Bioengineering, 8093 Zürich, Switzerland;

[6]EYE-Lab, Institute of Applied Sciences and Intelligent Systems, National Research Council, Via Pietro Castellino 111, 80131 Napoli, Italy;

[7]European Spallation Source ERIC, P.O. Box 176, SE 22100, Lund, Sweden.

[8] Institut Laue-Langevin, 71 Avenue des Martyrs, CS20156, 38042 Grenoble, France.

*Corresponding authors:

jacopo.cardellini@unifi.it

debora.berti@unifi.it

Table S1: *Hydrodynamic diameter (D_h) and polydispersity index (PDI) (obtained by cumulant analysis of DLS autocorrelation functions), ζ-potential, lattice parameter (d), and water channel radius (r_w) of GMO and GMO(SM) LCNPs. See methods section of main text for r_w calculation.*

	D_h (nm)	PDI	ζ-pot (mV)	d (nm)	r_w (nm)
GMO	255	0.25	-21 ± 2	14.3 ± 0.1	2.6 ± 0.1
GMO(SM)	270	0.24	5 ± 2	30-80	5.2 ± 0.8

Table S2: *Characteristic literature values of density, size, and lipid composition of human plasma lipoproteins (readapted from[1]).*

	HDL	LDL	VLDL
Density (g/mL)	1.06-1.21	1.02-1.06	0.96-1.01
Diameter (nm)	5-12	15-25	30-80
Triglycerides (%w/w)	2-7	4-8	45-65
Phospholipid (%w/w)	26-32	18-34	15-20
Cholesteryl ester (%w/w)	15-20	45-50	6-10
Cholesterol (%w/w)	3-5	6-8	4-8

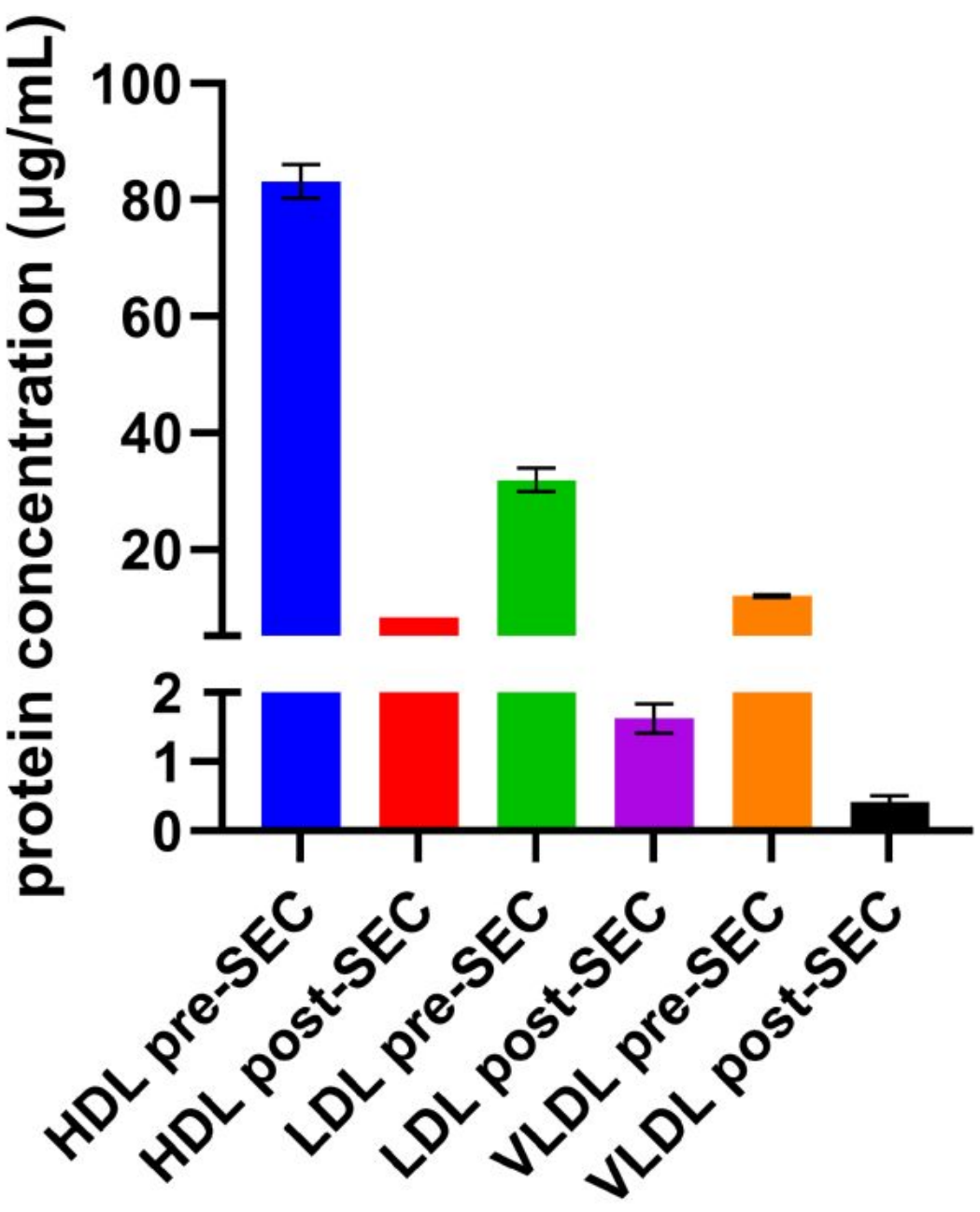

Figure S1: *Protein concentration in LDL, HDL, and VLDL before and after purification with SEC calculated using a Pierce Micro BCA™ Protein Assay kit (Thermo Fisher Scientific, MA, US), following the "Test Tube Procedure" indicated on the manufacturer's protocol. Data used to calculate lipoprotein dilution after SEC purification from Alexa-Flour 647 NHS.*

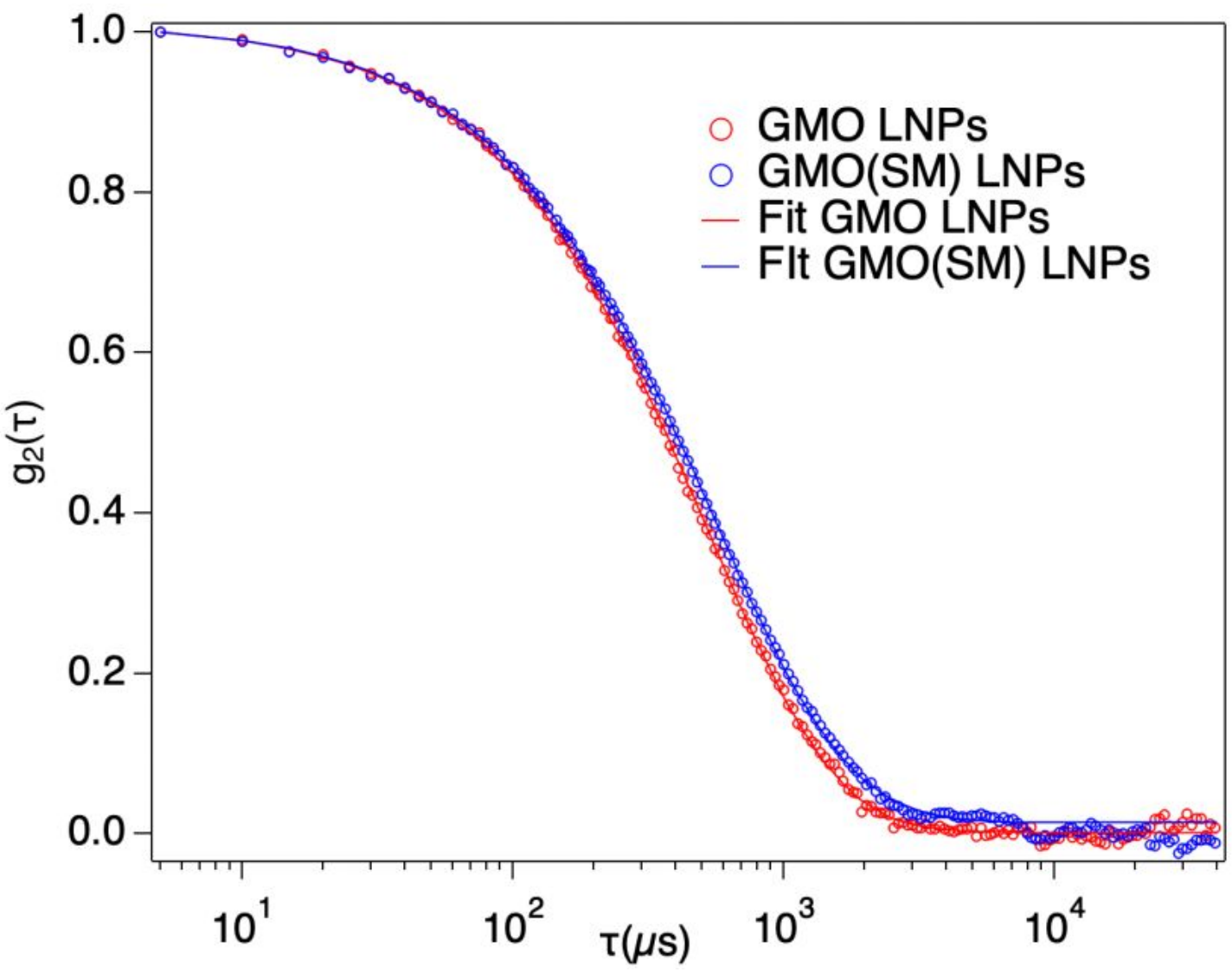

Figure S2: *Normalized DLS autocorrelation functions of GMO LNPs (red) and GMO(SM) LNPs (blue) in Tris buffer, 10 mM, pH 7.5 ± 0.1, analyzed with cumulant fitting.*

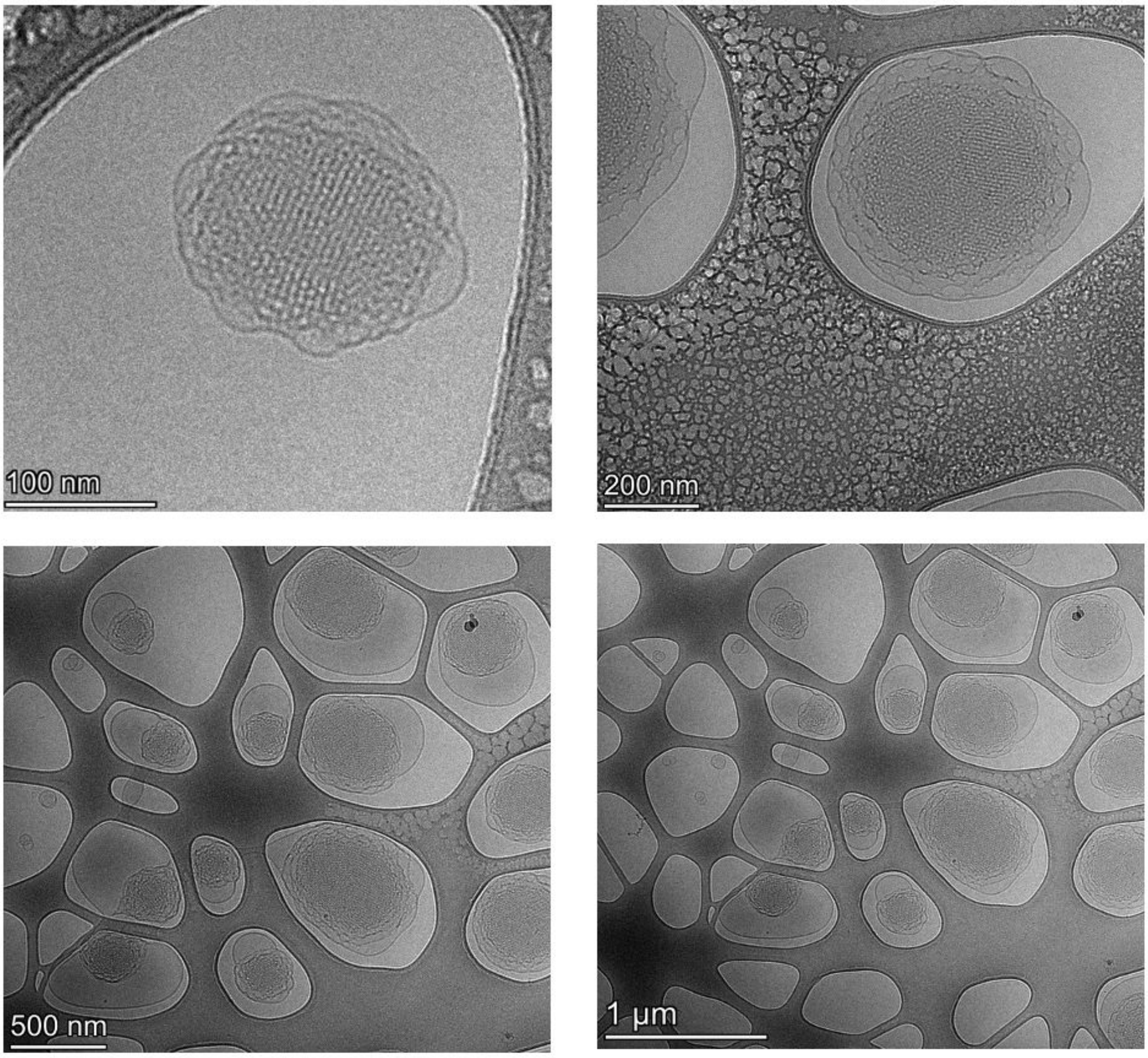

Figure S3: *Representative Cryo-TEM micrographs of GMO LCNPs in Tris buffer, 10 mM, pH 7.5 ± 0.1 at different magnifications.*

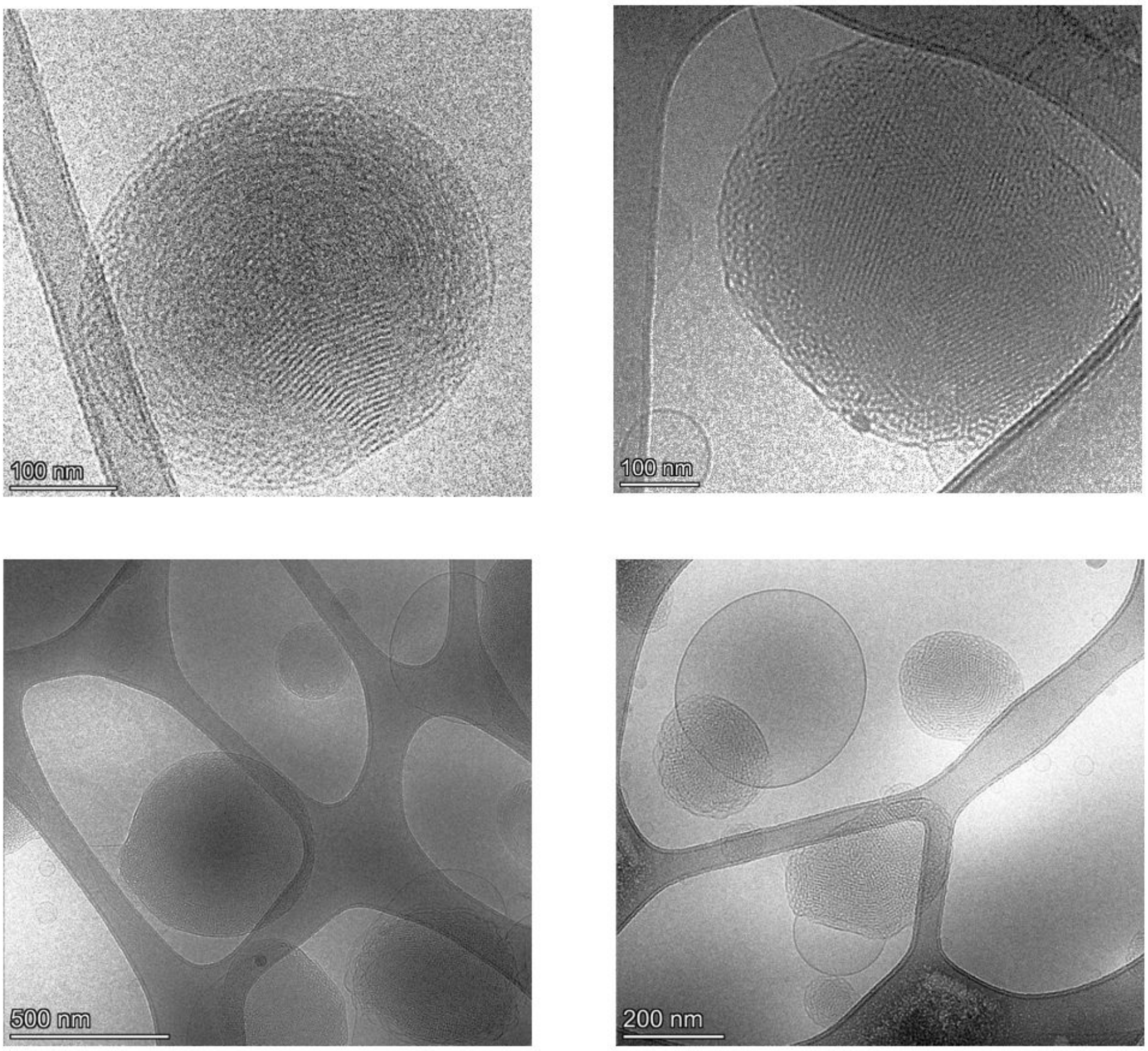

Figure S4: *Representative Cryo-TEM micrographs of GMO(SM) LCNPs in Tris buffer, 10 mM, pH 7.5 ± 0.1 at different magnifications.*

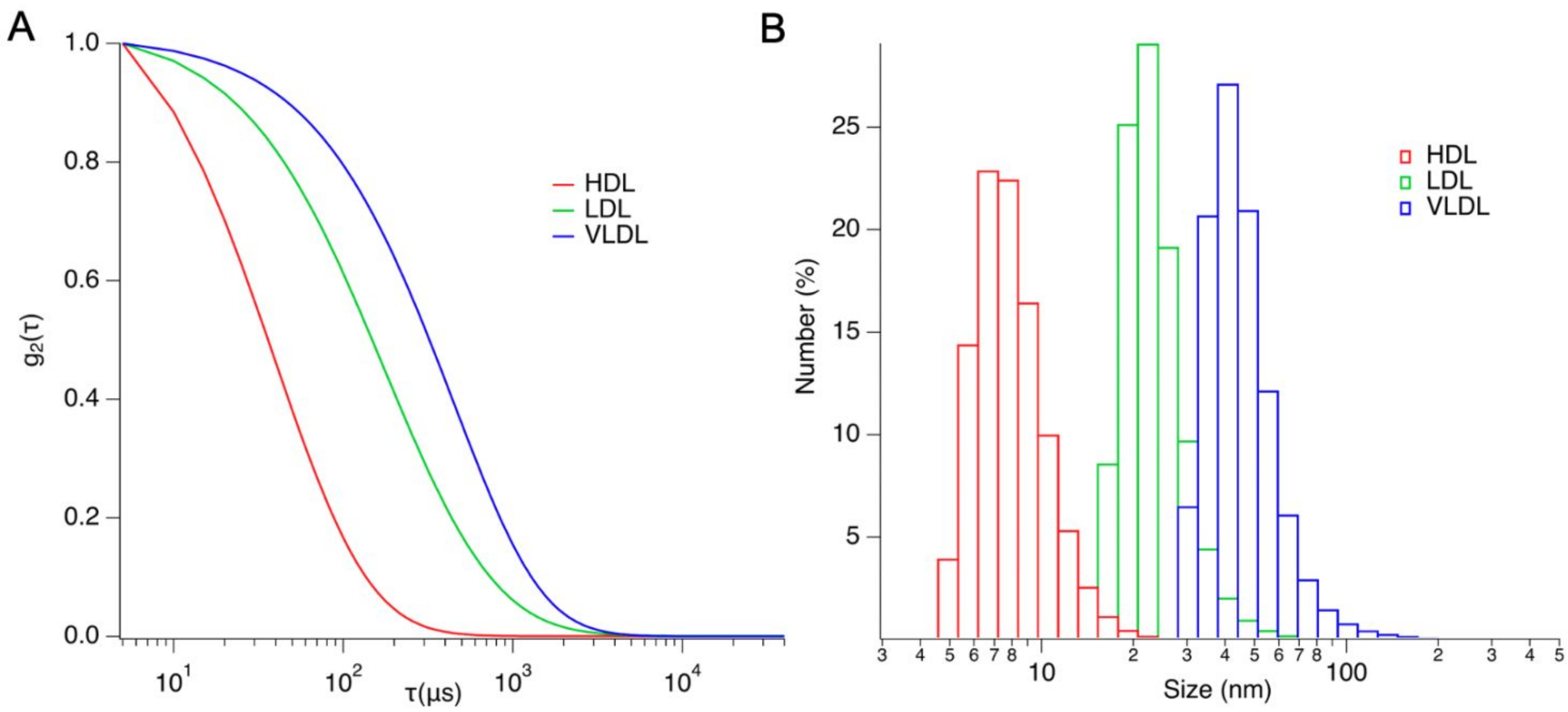

Figure S5: *Normalized DLS autocorrelation functions (A) and number-weighted size distribution (B) of HDL (red), LDL (green), and VLDL (blue) in Tris buffer, 10 mM, pH 7.5 ± 0.1. Number-weighted size distribution obtained by Contin fitting. number-weighted*

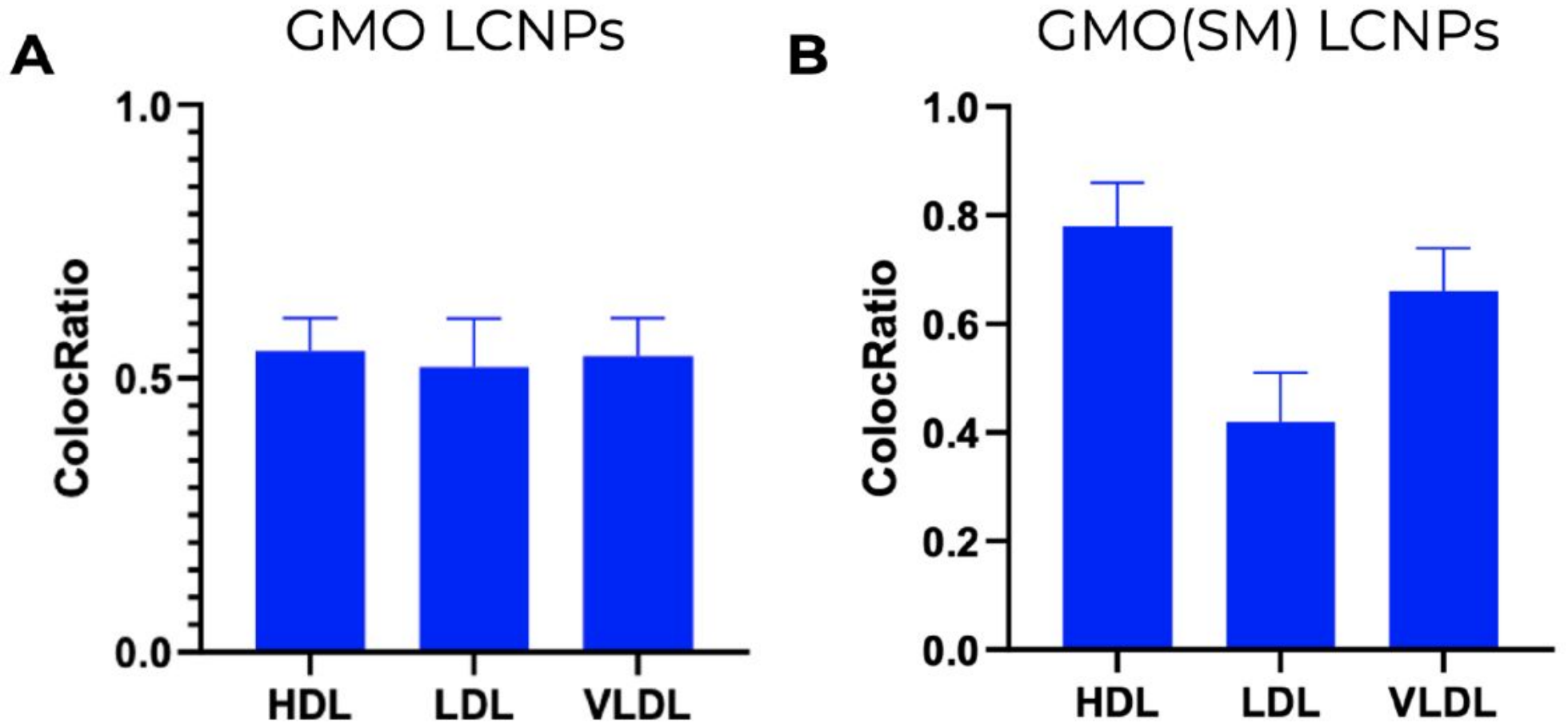

Figure S6: *Colocalization ratio obtained from fluorescence NTA measurements of BODIPY-labelled (A) GMO and (B) GMO(SM) LCNPs after 2 h incubation with Alexa Fluor 647-labelled a single class of lipoproteins (HDL, LDL, or VLDL; lipoproteins/LCNPs 5 wt%).*

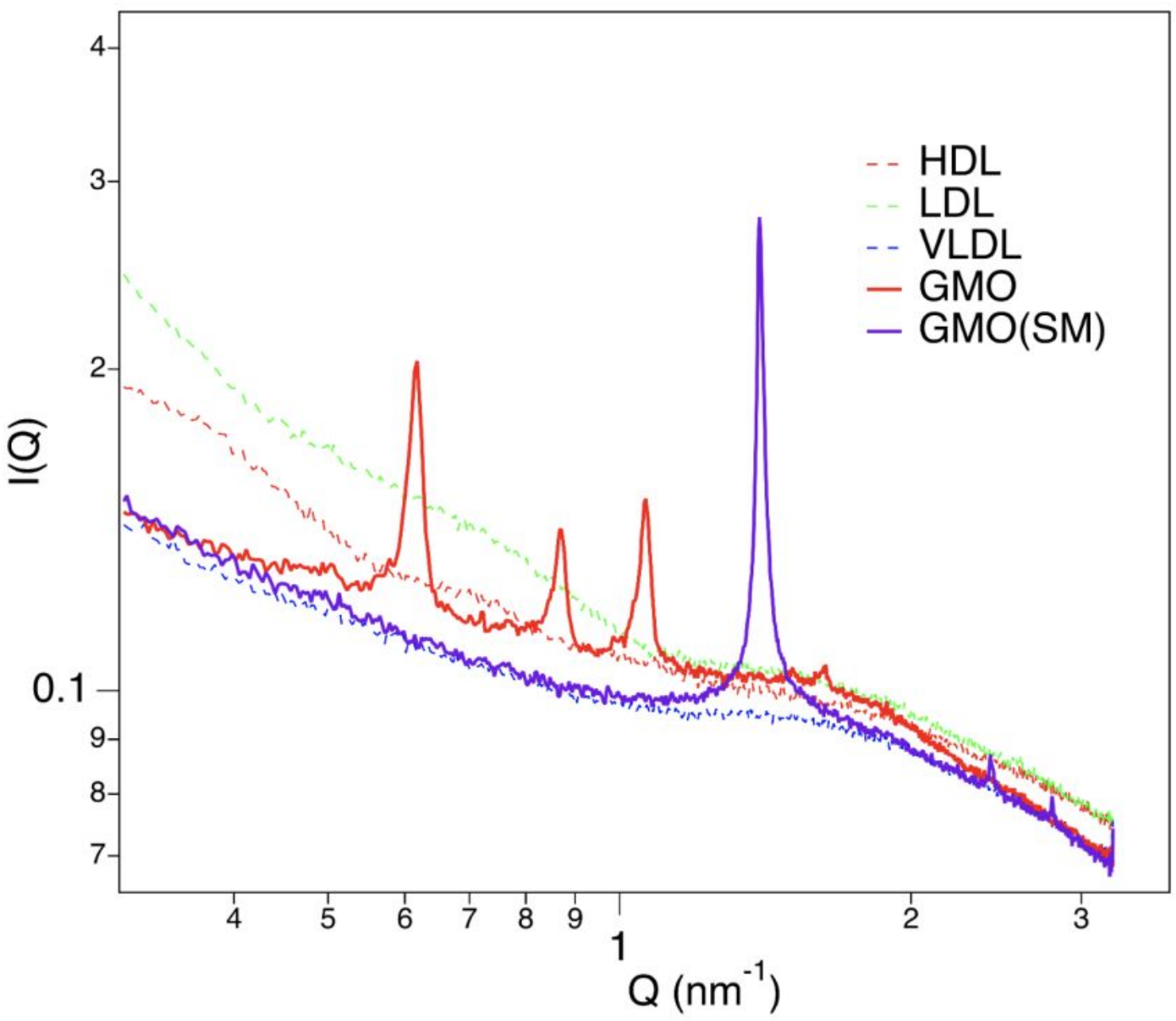

Figure S7: *SAXS profiles comparing GMO LCNPs and GMO(SM) LCNPs at 12 mg/mL to SAXS profiles of HDL, LDL, and VLDL at 0.5 mg/mL corresponding to Lipoproteins/LCNPs 5%wt. To allow reliable detection of GMO and GMO(SM) brag peaks, Lipoproteins/LCNPs 5%wt has been used as the highest quantity present in LCNPs-lipoprotein mixed formulations.*

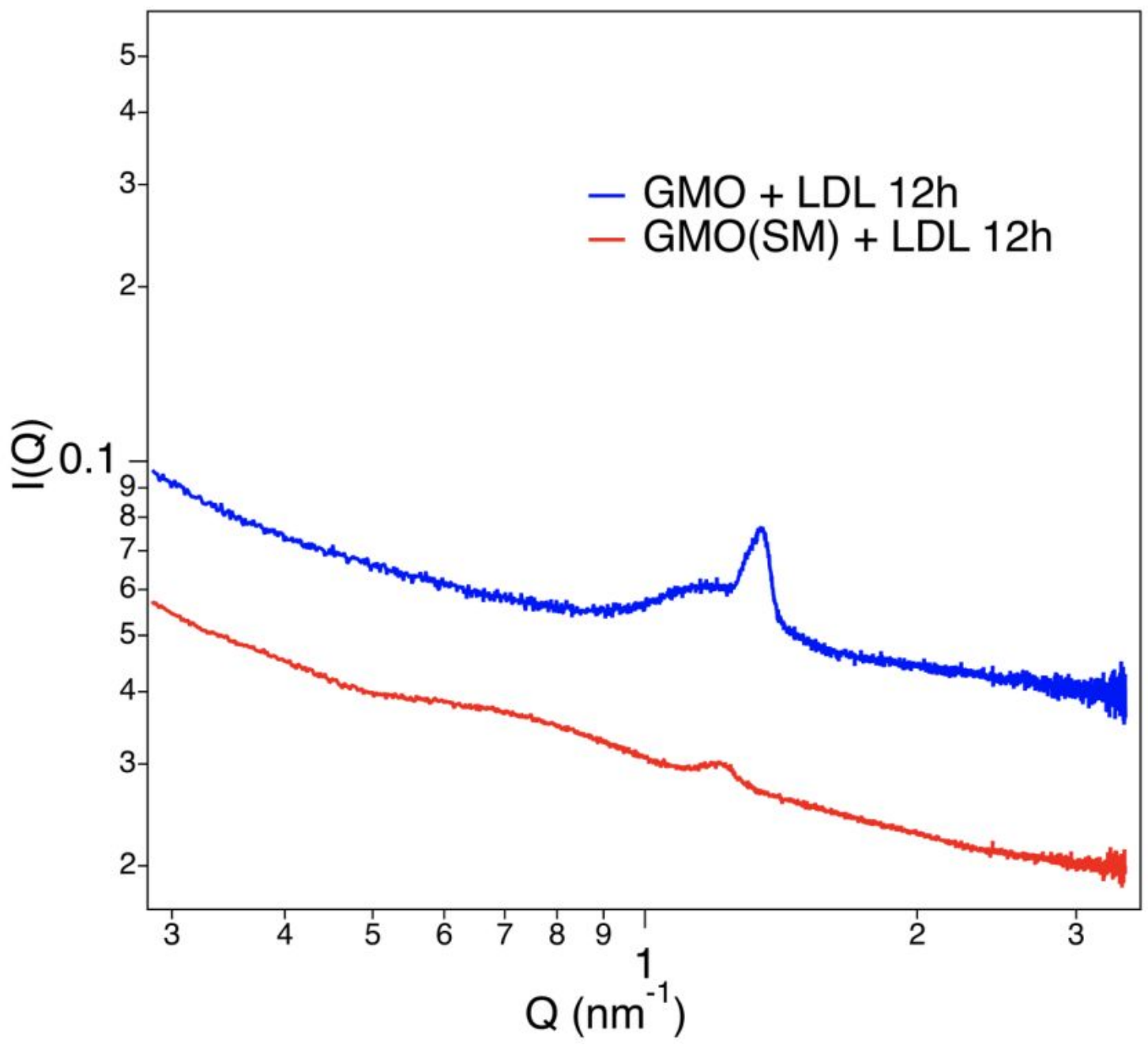

Figure S8: *SAXS profiles of GMO LCNPs (blue) and GMO(SM) (red) LCNPs at 12.5 mg/mL mixed with LDL, at 0.4 mg/mL measured after 12h of incubation (LCNPs/LDL 5%wt). After 12h of interaction, the original Bragg peaks of the GMO Im3m phase have completely vanished. The principal Bragg peak of the H_{II} phase of GMO(SM) LNPs is still clearly present but superimposed with an additional broad peak, highlighting the increased structural disorder.*

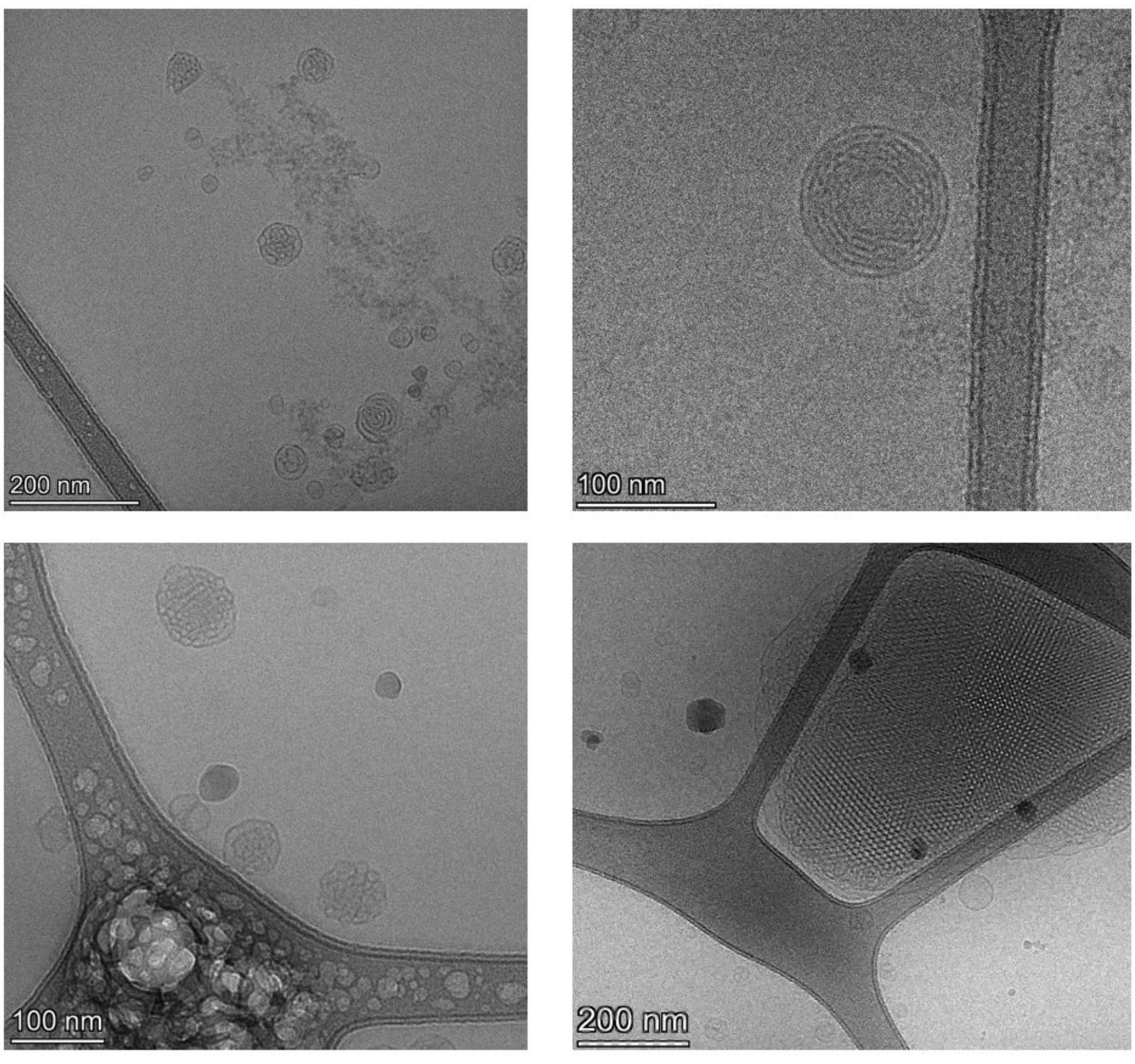

Figure S9: *Cryo-TEM images of GMO LCNPs after 2 h of interaction with LDL at an LDL/LCNP weight ratio of 5 wt% collected at different magnifications, showing the coexistence of sponge-like, cubic, amorphous, and multilamellar LNPs.*

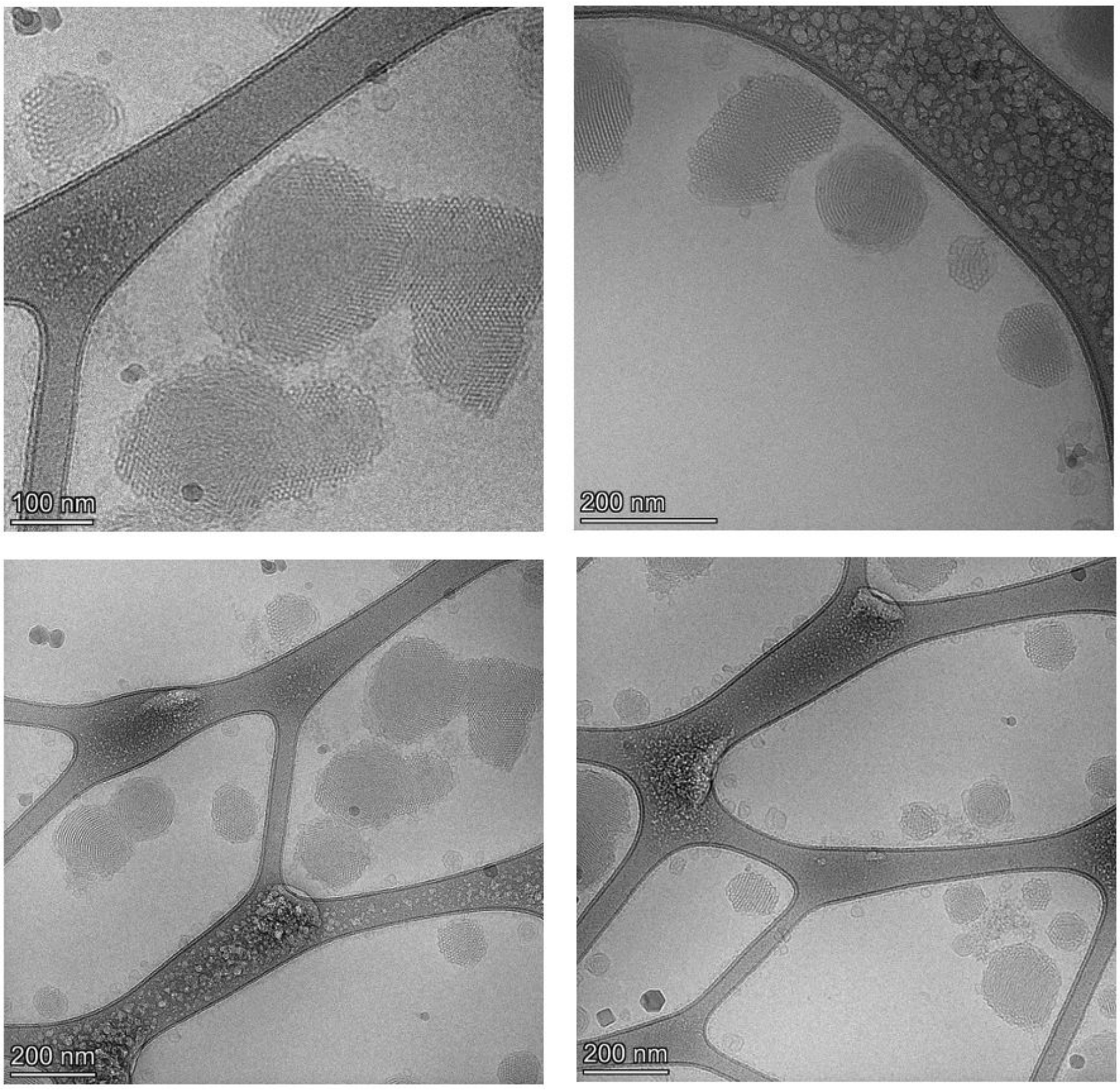

Figure S10: *Cryo-TEM images of GMO(SM) LCNPs after 2 h of interaction with LDL at an LDL/LCNP weight ratio of 5 wt% collected at different magnifications, showing the coexistence of sponge-like, cubic, amorphous, and multilamellar LNPs.*

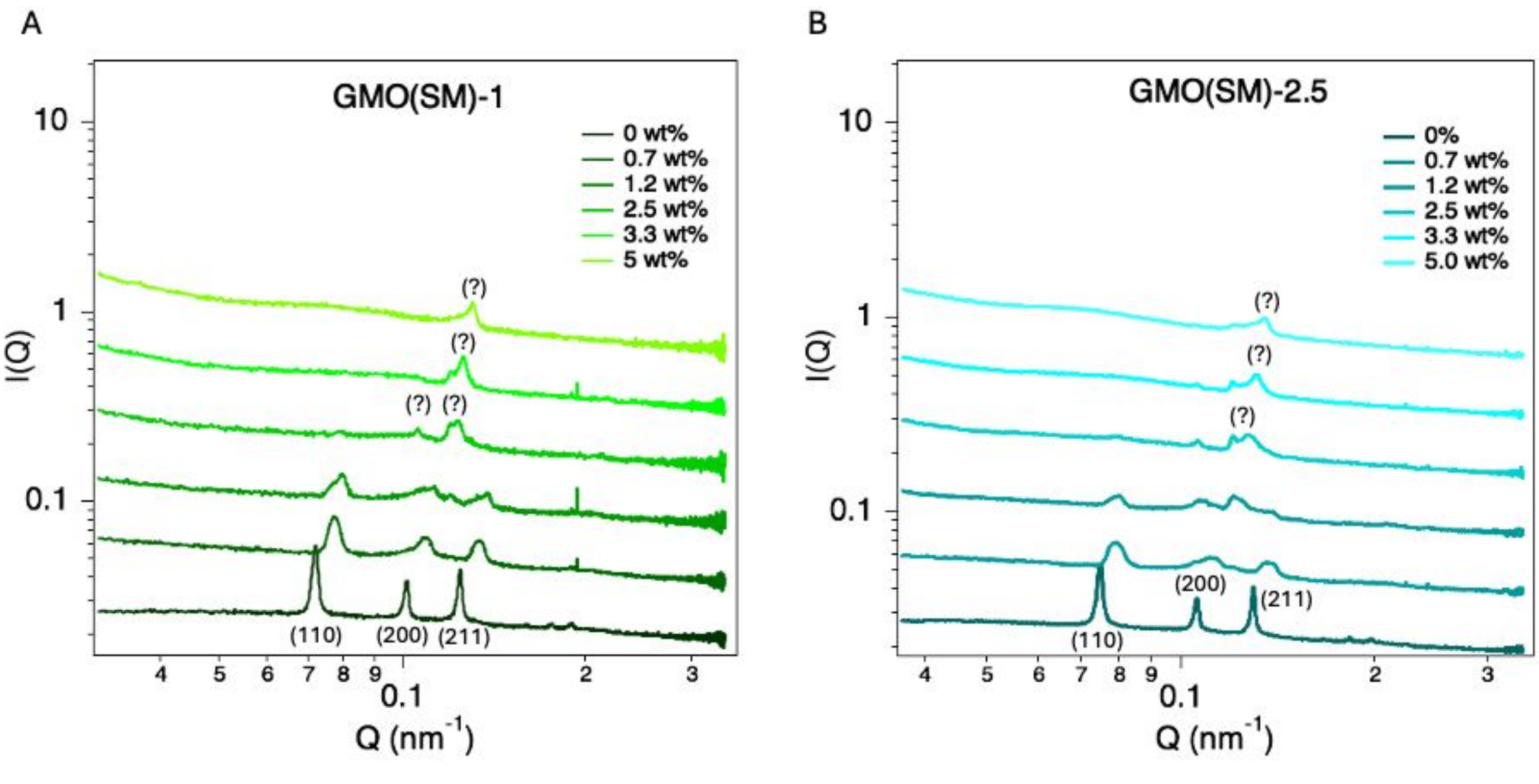

Figure S11: *SAXS profiles of (A) GMO(SM)-1 LCNPs containing GMO/SM-102 at a 99:1 molar ratio and (B) GMO(SM)-2.5 LCNPs containing GMO/SM-102 at a 97.5:2.5 molar ratio, after 2 h of incubation with LDL at LDL/LCNP weight ratios of 0, 0.7, 1.2, 2.5, 3.3, and 5 wt%. The LCNP concentration was maintained at 12.5 mg mL⁻¹. Data were acquired at beamline B21 of Diamond Light Source. The profiles are vertically offset for clarity. All measurements were performed in 10 mM Tris buffer, pH 7.5 ± 0.1, at 25 °C.*

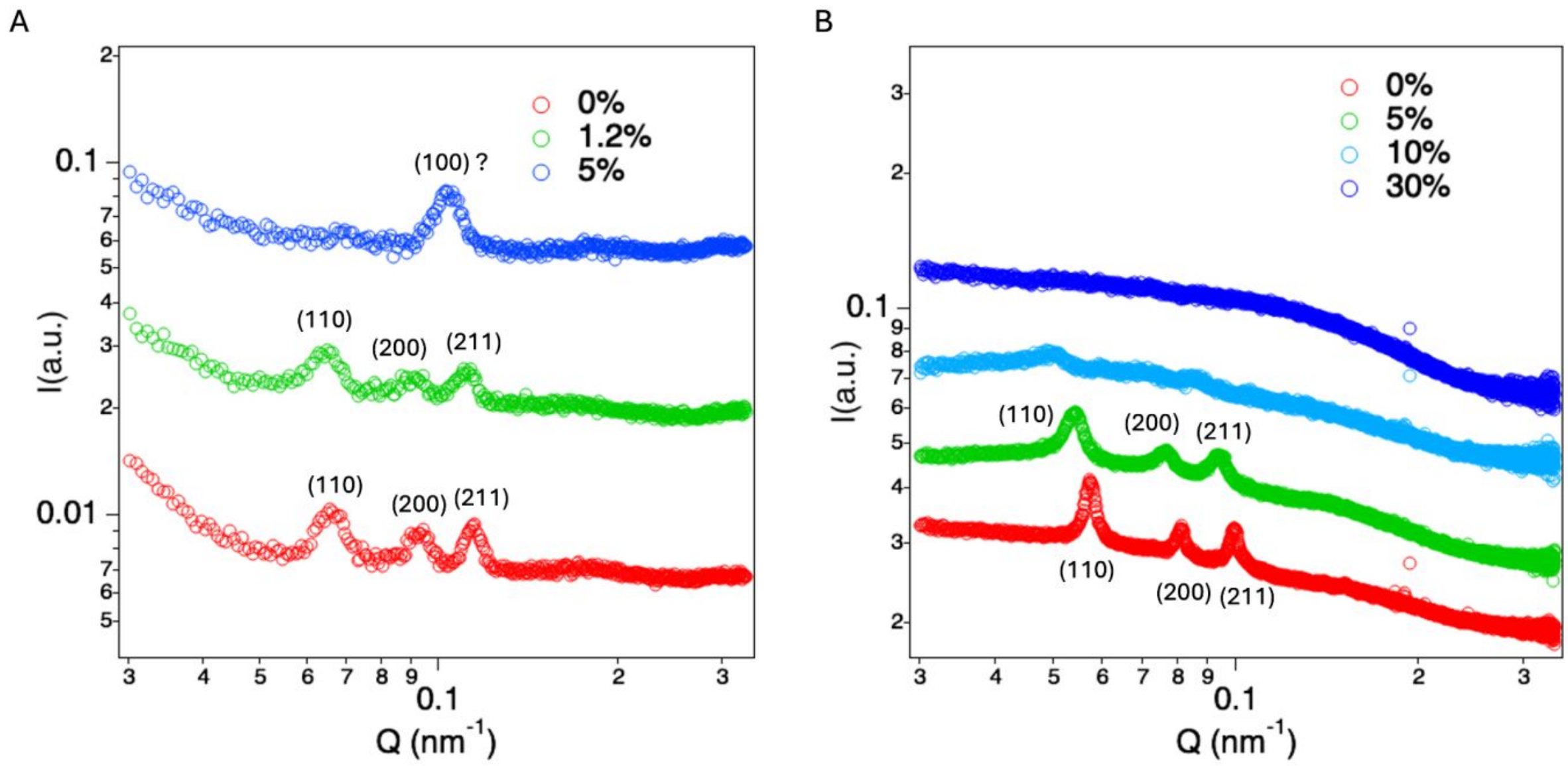

Figure S12: *(A) SAXS profiles of GMO LCNPs (12.5 mg mL⁻¹) after 2 h of incubation with protein-free LDL-inspired lipid mimics at mimic/LCNP weight ratios of 0, 1.2, and 5 wt%. The mimics contained POPC (1-palmitoyl-2-oleoyl-sn-glycero-3-phosphocholine), triolein, cholesteryl oleate, and cholesterol in a 6:26:48:7 mass ratio. The lipid composition of LDL mimics was selected based on literature review[1] (see also Table S2 for details on the composition of biological LDL). Data were acquired using an in-house Xenocs XEUSS 3.0 SAXS instrument. (B) SAXS profiles of GMO LCNPs containing 0, 5, 10, and 30 wt% cholesterol, acquired at beamline B21 of Diamond Light Source. The profiles are vertically offset for clarity. All measurements were performed in 10 mM Tris buffer, pH 7.5 ± 0.1, at 25 °C.*

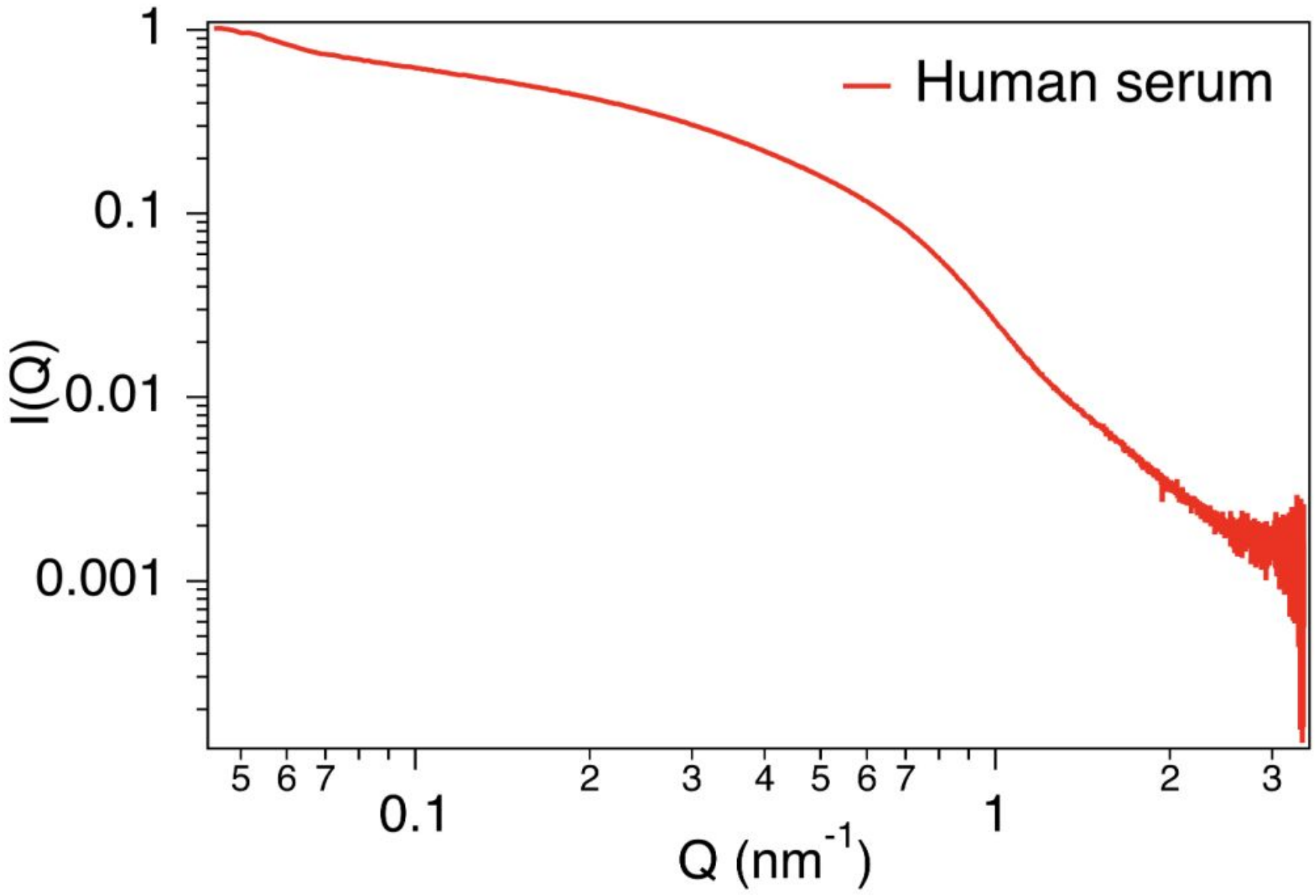

Figure S13: *SAXS curve of human serum showing the characteristic profile of globular proteins highly abundant in serum.*

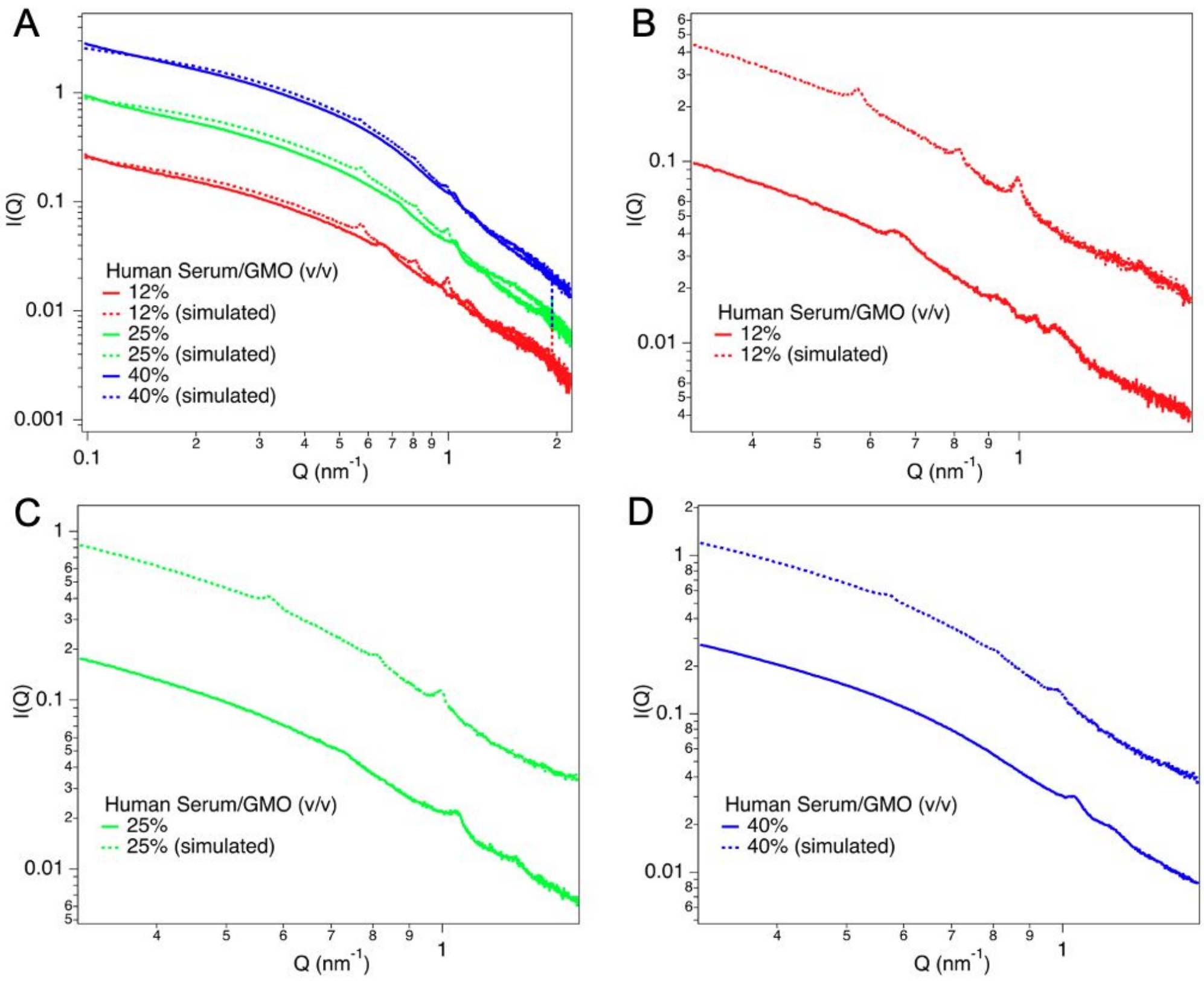

Figure S14: *SAXS profiles of GMO LCNPs mixed with different volumes of human serum (continuous lines) compared to linear sum of SAXS profiles of GMO LCNPs and human serum (dashed lines).*

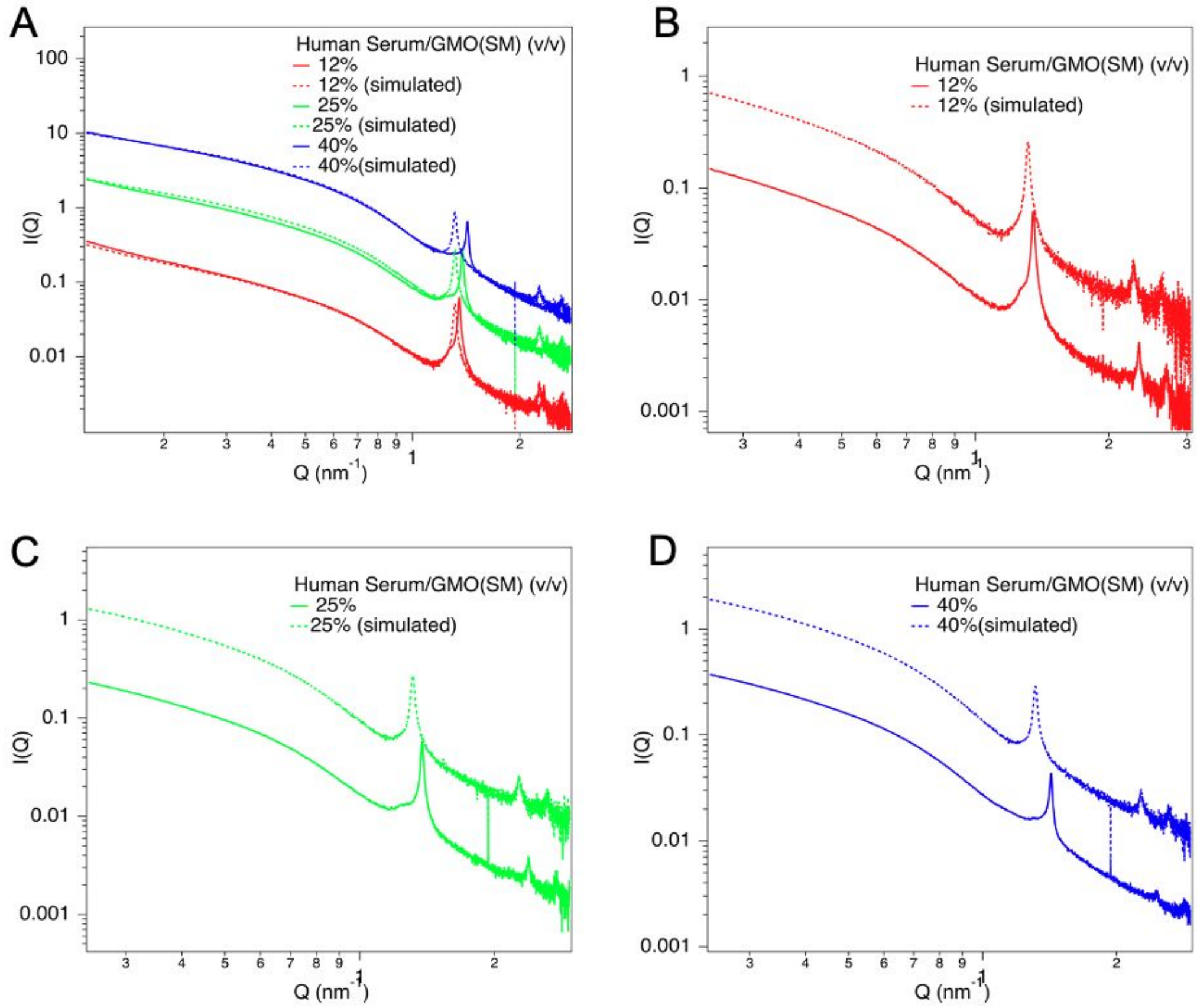

Figure S15: *SAXS profiles of GMO (SM) LCNPs mixed with different concentrations of human serum (continuous lines) compared to linear sum of SAXS profiles of GMO(SM) LCNPs and human serum (dashed lines).*

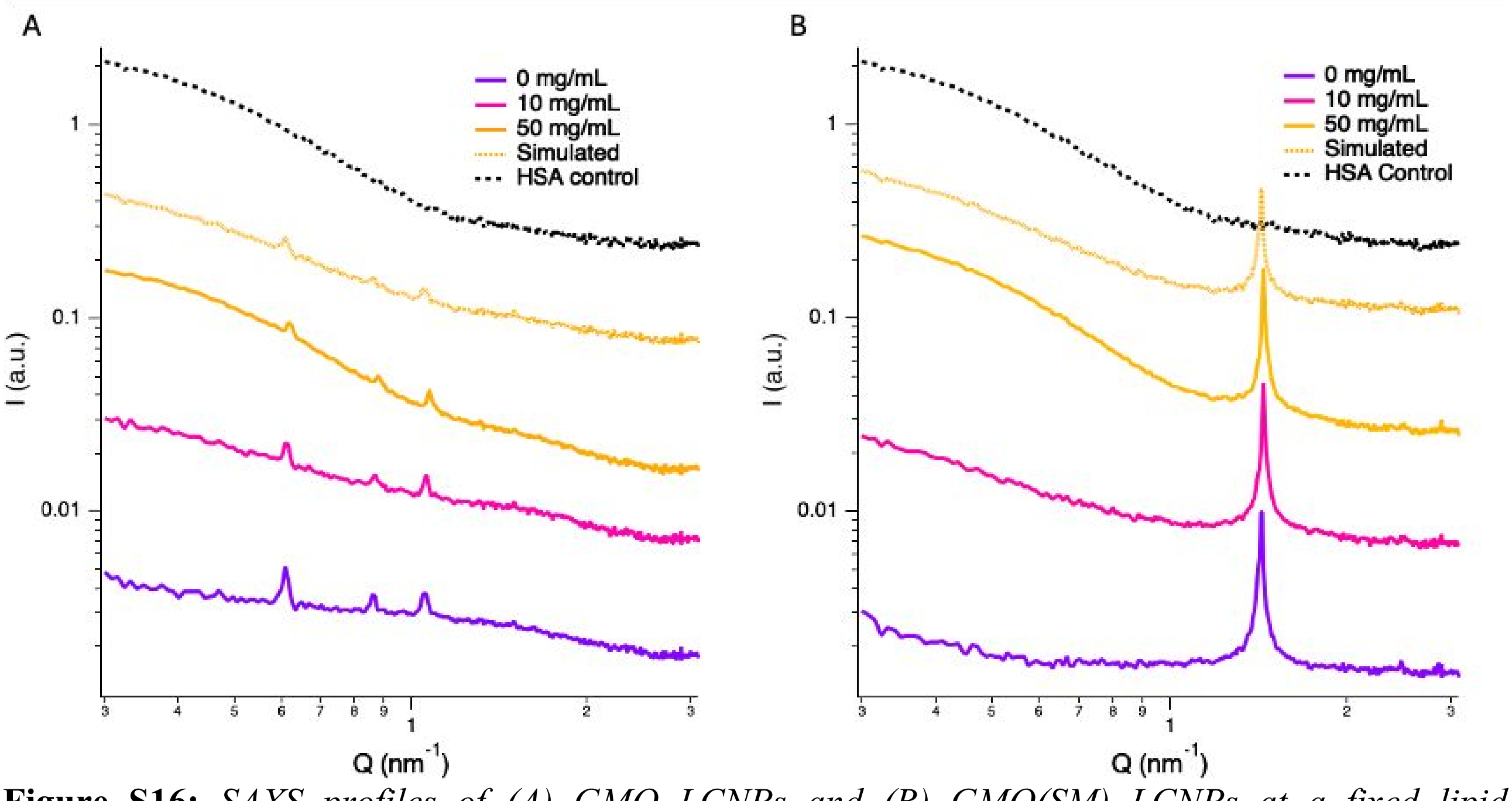

Figure S16: *SAXS profiles of (A) GMO LCNPs and (B) GMO(SM) LCNPs at a fixed lipid concentration of 12.5 mg mL* $^{-1}$ *after 2 h of incubation with HSA at concentrations ranging from 0 to 50 mg mL* $^{-1}$*. The profile of HSA alone at 50 mg mL* $^{-1}$ *and the corresponding simulated non-interacting profile obtained by linear combination of the separately measured LCNP and HSA scattering curves are included as controls. Data was acquired at beamline B21 of Diamond Light Source. The profiles*

are vertically offset for clarity. All measurements were performed in a 10 mM Tris buffer, pH 7.5 ± 0.1, at 25 °C.

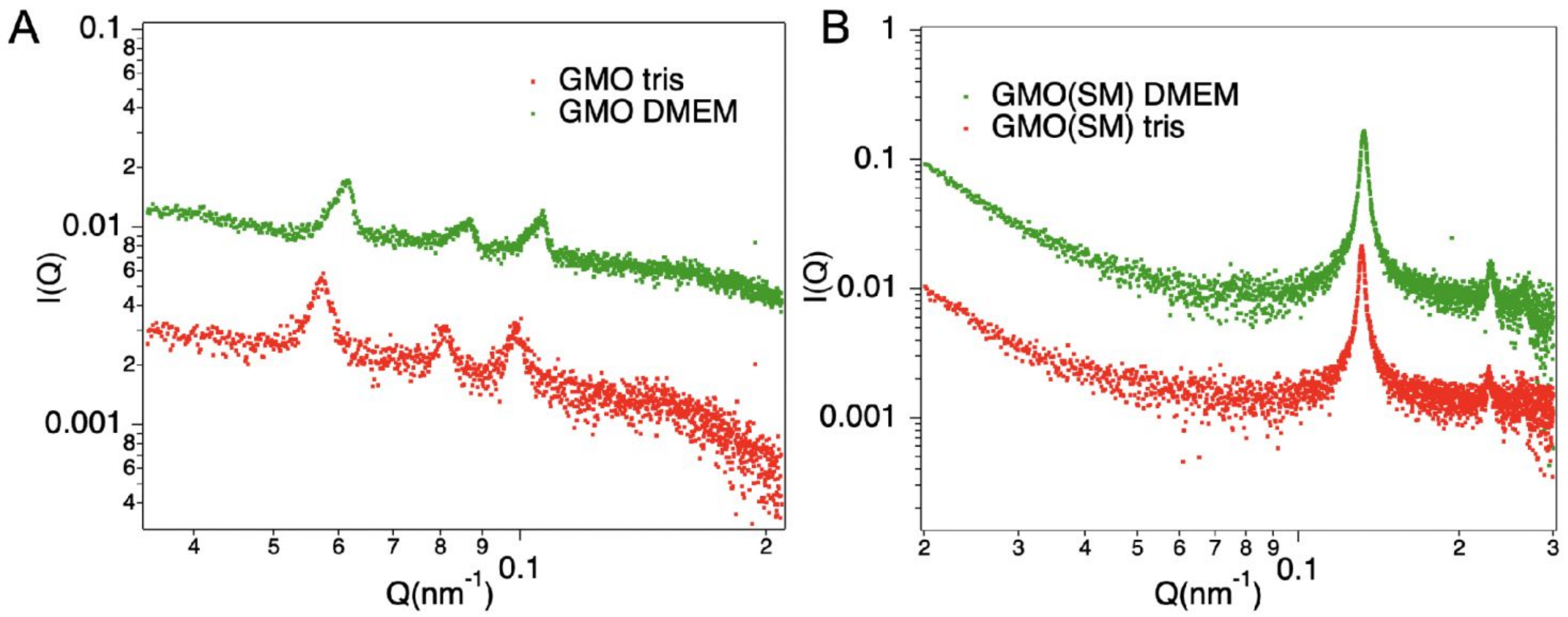

Figure S17: *SAXS profiles of A) GMO LCNPs and B) GMO(SM) LCNPs diluted in DMEM (LCNPs 3 mg/mL) compared to LCNPs in Tris 10 mM pH = 7.5, showing no significant effect of DMEM (cell medium) on LCNP structure.*

Synthesis of d-GMO

General. Reagents were purchased from commercial suppliers and used without further purification. Flash chromatography was carried out using silica gel (40 – 63 µm, 230 – 400 mesh). NMR spectra were recorded on a Bruker AVANCE spectrometer (500 and 126 MHz for ^{1}H and ^{13}C NMR respectively).

(2,2-dimethyl-1,3-dioxolan-4-yl)methyl oleate-d$_{32}$. To a solution of DL-isopropylidene glycerol (420 mg, 3.2 mmol), oleic acid-d$_{32}$ (1g, 3.2 mmol) and DMAP (408 mg, 3.3 mmol) in anhydrous DCM (75 mL) was added DCC (1M in DCM, 4.8 mL, 4.8 mmol) at r.t. under N_2. After stirring for 2 days, the reaction mixture was filtered, rinsed with DCM (75 mL) and evaporated under reduced pressure. The residue was purified by column chromatography using hexane/EtOAc (92:8) as eluent to afford the title compound as colourless oil (1g, 75%). ^{1}H NMR (500 MHz, $CDCl_3$) δ 5.30 (s, 1H), 4.29 (m, 1H), 4.14 (ddd, J = 11.5, 5.0 ,1.5 Hz, 1H), 4.09 – 4.04 (complex, 2H), 3.72 (m, 1H), 2.28 (m, 0.12H*), 1.94 (m, 0.14H*), 1.73 (m, 0.1H*), 1.41 (s, 3H), 1.35 (s, 3H), 1.25 – 1.17 (complex, 0.87H*), 0.80 (m, 0.12H*). *Contribution from residual protium. ^{13}C NMR (126 MHz, $CDCl_3$) δ173.6, 129.5 (m), 129.4, 109.7, 73.6, 66.3, 64.4, 33.4 (m), 30.7 (m), 29.0 – 27.6 (complex), 26.6, 26.4 – 25.8 (complex), 25.3, 24.3 – 23.6 (complex), 21.9 – 21.1 (complex), 12.9 (m).

Glyceryl monooleate-d$_{32}$. To a solution of (2,2-dimethyl-1,3-dioxolan-4-yl)methyl oleate-d$_{32}$ (900 mg, 2.1 mmol) in ethanol/water (25 mL, 95:5) was added Amberlite resin (4.4 g), which has been prewashed with methanol (3 x 20 mL) and ethanol (3 x 20 mL), at r.t. under N_2. After stirring at 90°C for 2 h, the reaction mixture was filtered, rinsed with ethanol (20 mL) and evaporated under reduced pressure. The residue was purified by column chromatography using DCM/EtOAc (100:0 □ 80:20) as eluent to afford the title compound as white solid (667 mg, 82%). ^{1}H NMR (500 MHz, CDCl3) δ 5.33 (s, 1H), 4.21 (ddd, J = 11.5, 4.5, 1.0 Hz, 1H), 4.15 (dd, J = 11.5, 6.0 Hz, 1H), 3.93 (m, 1H), 3.70 (m, 1H), 3.60 (m, 1H), 2.48 (s, 1H), 2.31 (m, 0.11H*), 2.04 (s, 1H), 1.96 (m, 0.14H*), 1.27 – 1.19 (complex, 0.88H*), 0.82 (m, 0.11H*). *Contribution from residual protium. ^{13}C NMR (126 MHz,

$CDCl_3$) δ 174.4, 129.5 (m), 129.4, 70.2, 65.0, 63.3, 33.4 (m), 31.0 – 30.3 (complex), 29.0 – 27.6 (complex), 26.6 – 25.8 (complex), 24.1 – 23.6 (complex), 21.9 – 21.1 (complex), 13.0 (m).

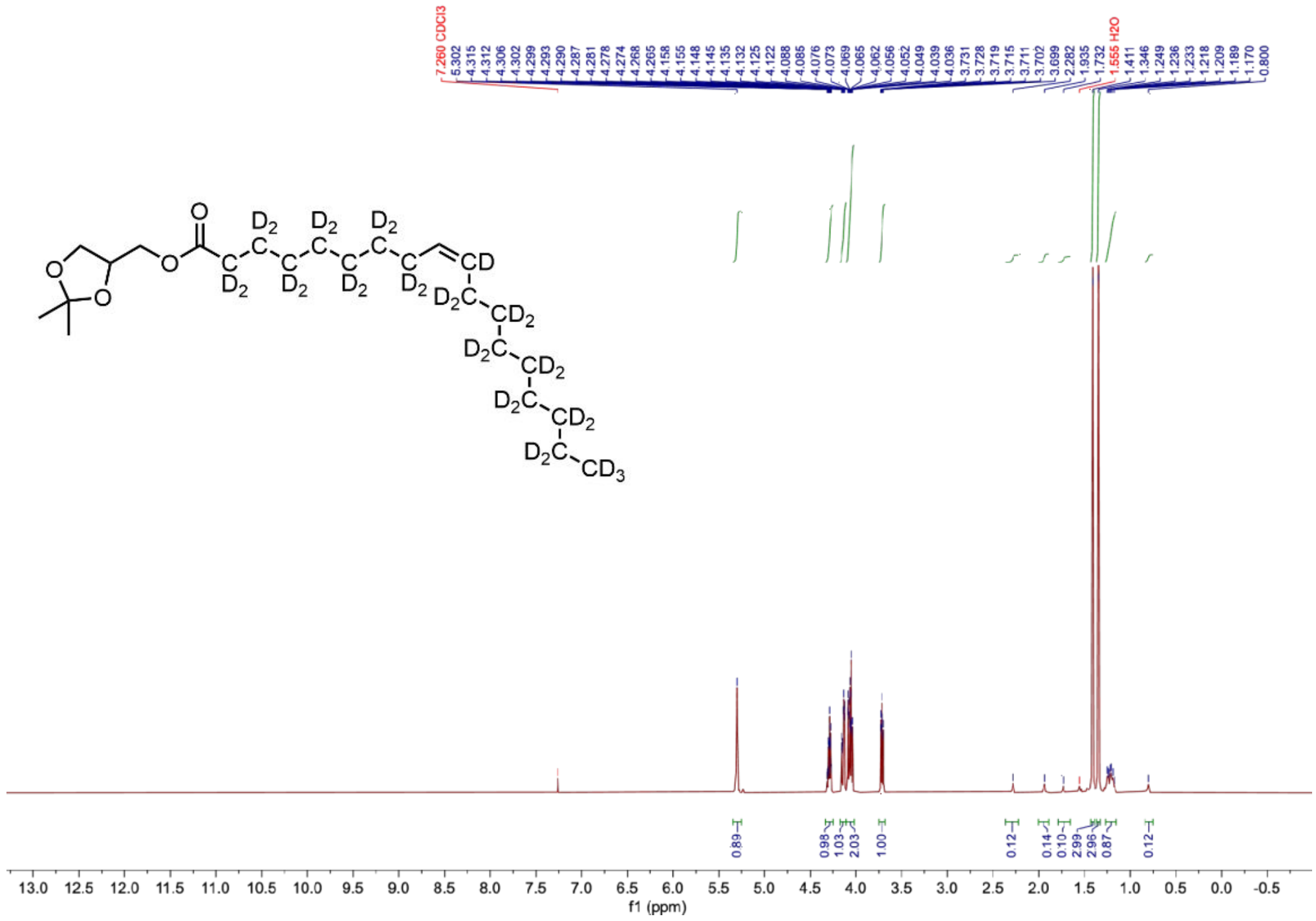

Figure S18. ^{1}H NMR spectrum of (2,2-dimethyl-1,3-dioxolan-4-yl)methyl oleate-d_{32}.

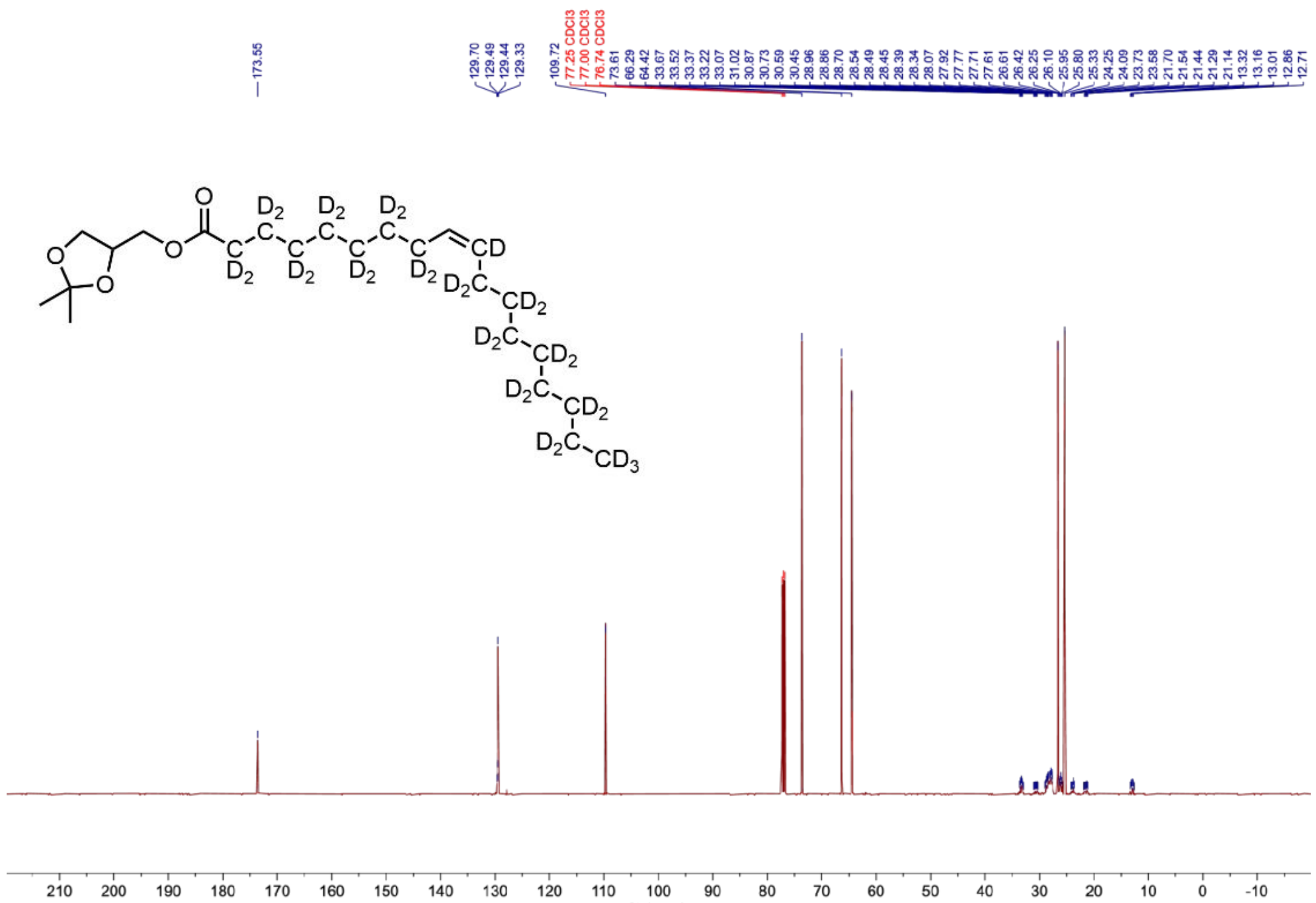

Figure S19. ^{13}C NMR spectrum of (2,2-dimethyl-1,3-dioxolan-4-yl)methyl oleate-d_{32}.

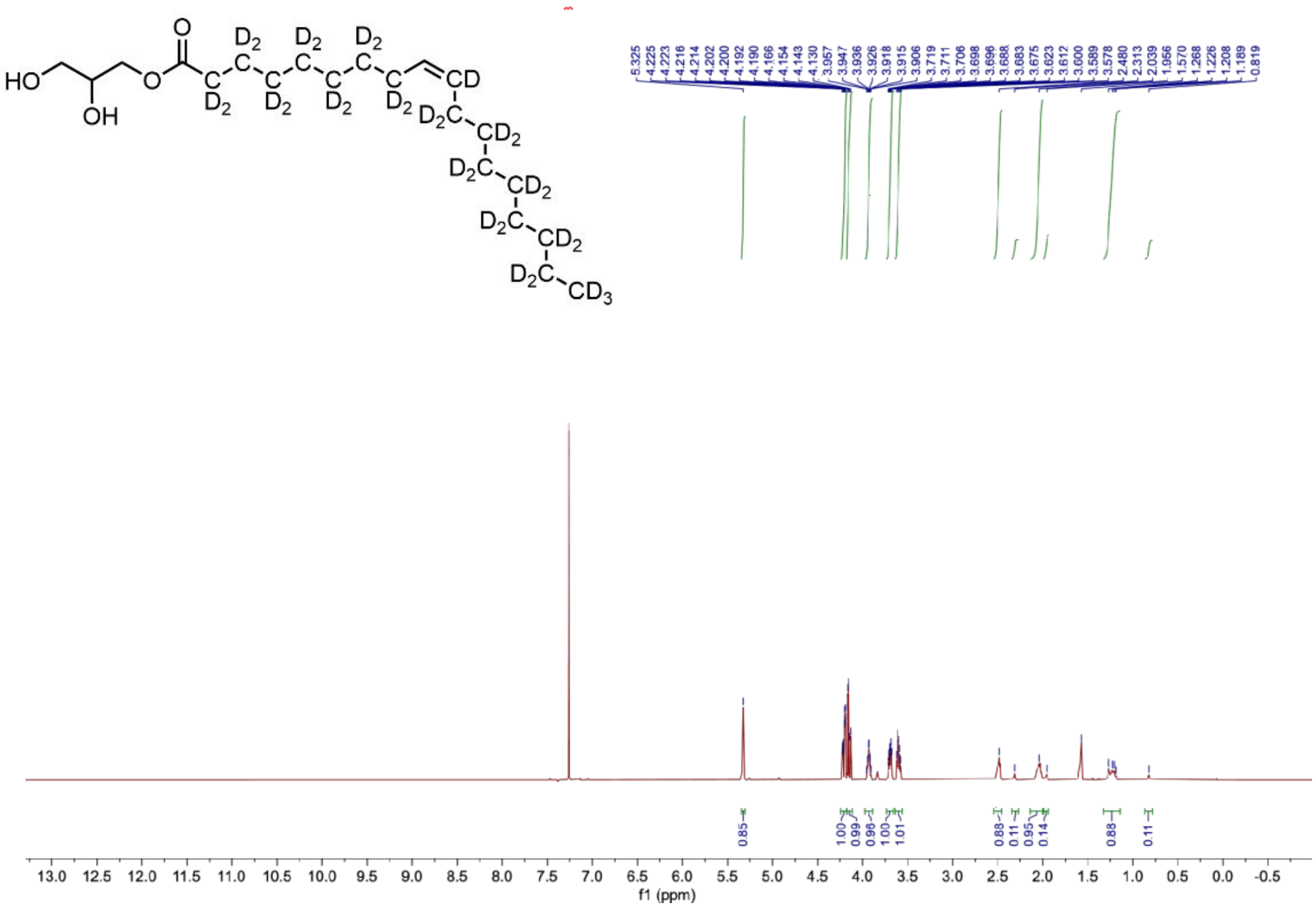

Figure S18. ^{1}H NMR spectrum of glyceryl monooleate-d_{32}.

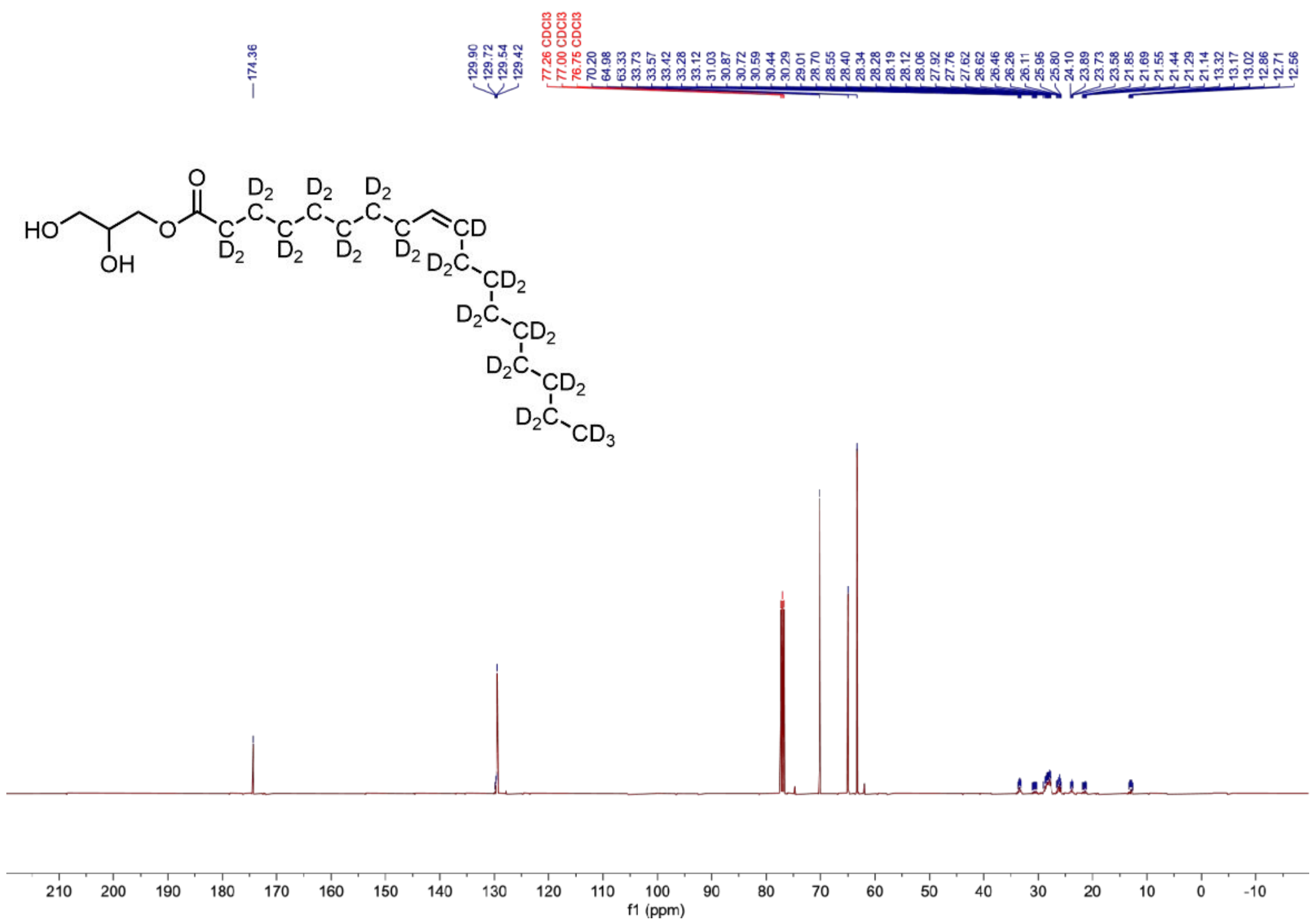

Figure S19. ^{13}C NMR spectrum of glyceryl monooleate-d_{32}.

References

(1) Rensen, P. C. N.; De Vrueh, R. L. A.; Kuiper, J.; Bijsterbosch, M. K.; Biessen, E. A. L.; Van Berkel, T. J. C. Recombinant Lipoproteins: Lipoprotein-like Lipid Particles for Drug Targeting. *Adv. Drug Deliv. Rev.* **2001**, *47* (2–3), 251–276. https://doi.org/10.1016/S0169-409X(01)00109-0.
